# Mycn ameliorates cardiac hypertrophy-induced heart failure in mice by mediating the USP2/JUP/Akt/β-catenin cascade

**DOI:** 10.1186/s12872-024-03748-8

**Published:** 2024-01-31

**Authors:** Weinian Gao, Na Guo, Hongjiang Yan, Shuguang Zhao, Yongquan Sun, Ziying Chen

**Affiliations:** 1 Department of Cardiac Surgery, The Second Hospital of Hebei Medical University, Shijiazhuang, Hebei 050000 P.R. China; 2 Hebei Medical University, Shijiazhuang, Hebei 050000 P.R. China; 3 Department of Geriatry II, TCM Hospital of Shijiazhuang city, Shijiazhuang, Hebei 050000 P.R. China; 4 Department of Thoracic surgery, The Second Hospital of Hebei Medical University, Shijiazhuang, Hebei 050000 P.R. China

**Keywords:** Cardiac hypertrophy, Heart failure, Mycn, USP2, JUP, Akt/β-catenin

## Abstract

**Background:**

Pathological cardiac hypertrophy is associated with cardiac dysfunction and is a key risk factor for heart failure and even sudden death. This study investigates the function of Mycn in cardiac hypertrophy and explores the interacting molecules.

**Methods:**

A mouse model of cardiac hypertrophy was induced by isoproterenol (ISO). The cardiac dysfunction was assessed by the heart weight-to-body weight ratio (HW/BW), echocardiography assessment, pathological staining, biomarker detection, and cell apoptosis. Transcriptome alteration in cardiac hypertrophy was analyzed by bioinformatics analysis. Gain- or loss-of-function studies of MYCN proto-oncogene (Mycn), ubiquitin specific peptidase 2 (USP2), and junction plakoglobin (JUP) were performed. The biological functions of Mycn were further examined in ISO-treated cardiomyocytes. The molecular interactions were verified by luciferase assay or immunoprecipitation assays.

**Results:**

Mycn was poorly expressed in ISO-treated mice, and its upregulation reduced HW/BW, cell surface area, oxidative stress, and inflammation while improving cardiac function of mice. It also reduced apoptosis of cardiomyocytes in mice and those in vitro induced by ISO. Mycn bound to the USP2 promoter to activate its transcription. USP2 overexpression exerted similar myocardial protective functions. It stabilized JUP protein by deubiquitination modification, which blocked the Akt/β-catenin pathway. Knockdown of JUP restored phosphorylation of Akt and β-catenin protein level, which negated the protective effects of USP2.

**Conclusion:**

This study demonstrates that Mycn activates USP2 transcription, which mediates ubiquitination and protein stabilization of JUP, thus inactivating the Akt/β-catenin axis and alleviating cardiac hypertrophy-induced heart failure.

**Supplementary Information:**

The online version contains supplementary material available at 10.1186/s12872-024-03748-8.

## Background

Mammalian heart muscle cells (cardiomyocytes) usually withdraw from the cell cycle shortly after birth, so most of these cells are terminally differentiated in adults and do not naturally replicate under normal circumstances [1]. Nevertheless, the heart tissue has the ability to adapt to changing conditions and can experience changes in size due to different stresses [2]. Consequently, when faced with internal or external triggers, the heart adjusts its dimensions and muscle mass through hypertrophic remodeling, aiming to bolster its contracting strength and overall workload capacity [3]. Although physiological hypertrophy can enhance cardiac performance and alleviate stress on the ventricular walls, instances of pathological hypertrophy occur due to prolonged stress or illness, leading to ventricular expansion, reduction in contraction capability, and eventually the development of heart failure [4, 5]. Despite identification of various mechanisms behind pathological hypertrophy, including malfunctioning mitochondria, cell death in heart tissue, and the generation of reactive oxygen species (ROS) [6], the molecular alterations responsible for these events remain largely unknown.

By conducting integrated transcriptome and enrichment analyses using publicly accessible datasets and tools, we obtained MYCN proto-oncogene (Mycn) as a promising gene whose low expression is potentially correlated with oxidative stress and progression of cardiac hypertrophy. The Mycn oncogene encodes the Mycn protein, a bHLH transcription factor that plays an essential role during embryonal development [7]. Mycn is a convergence point for multiple developmental signaling pathways, and it can influence cell growth, proliferation, differentiation, or apoptosis, depending on the cellular context [7, 8]. Interestingly, Mycn gene deletion in mouse myocardium has been found to lead to a thin-myocardial wall anomaly with a significant reduction in trabeculation, implying its crucial role in shaping the morphology of the myocardial wall [8]. However, the expression profile and biological functions of Mycn in pathological cardiac hypertrophic remain unclear. Furthermore, our bioinformatics analyses suggested ubiquitin specific peptidase 2 (USP2) as a promising target of Mycn and junction plakoglobin (JUP) a promising substrate of USP2. USP2 has recently been demonstrated to exert a myocardium-protective role in cardiac hypertrophy [9]. It is a multifunctional deubiquitinating enzyme (DUB) that has been associated with cell cycle progression and tumorigenesis [10]. As a member of the USP subfamily of DUB, USP2 potentiates the stability of target proteins by inhibiting proteasome-dependent protein degradation [10, 11]. Concerning JUP, eliminating it specifically from the heart of mice led to gradual cardiomyocyte loss, replacement of tissue with fibrous matter, extensive infiltration of inflammatory cells, and impairment of heart function, which was attributed to the Akt/β-catenin signaling activation [12]. Indeed, the Akt/β-catenin signaling has been closely associated with the pathology of cardiac hypertrophy [13]. Collectively, we hypothesized that Mycn possibly regulates USP2 transcription, which modulates JUP protein stability and the Akt/β-catenin signaling to affect cardiac hypertrophy progression.

## Materials and methods

### Animals

Four-week-old male C57BL/6 mice (18–22 g) were procured from Shanghai SLAC Laboratory Animal Co., Ltd. (Shanghai, China). They were housed in separated cages in temperature-controlled rooms (21 ± 2℃), in a 12/12-h light/dark cycle, and were provided with feed and water *ad libitum*. All animal study experiments were performed in adherence to the NIH Guidelines on the Use of Laboratory Animals and the ARRIVE guidelines and were approved by the Committee on Animal Care of Second Hospital of Hebei Medical University.

### A mouse model of hypertrophic cardiomyopathy induced by isoproterenol (ISO)

Hypertrophic cardiomyopathy (HCM) in mice was generated by the injection of ISO. In short, the mice were subcutaneously injected with ISO hydrochloride (1351005, Merck KGaA, Darfmstadt, Germany) for nine consecutive days (5 mg/kg/d). Mice in the sham group were injected with normal saline instead. After 9 d, the mice were weighed, followed by echocardiographic analysis. Subsequently, the mice were euthanized by intraperitoneal injection of an overdose of pentobarbital sodium (150 mg/kg) [14]. Successful animal death was confirmed by the loss of corneal and nerve reflexes with noticeable pupil dilation, the absence of respiration, pulse, and heartbeat for more than 5 min, and the possibility of simple cardiac arrest was excluded [15]. Subsequently, their hearts and blood samples were extracted. The heart weight-to-body weight ratio (HW/BW) was calculated.

For artificial gene interference in mice, the animals were injected with lentiviral vectors-packaged gene overexpression plasmids of Mycn (OE-Mycn), USP2 (OE-USP2), gene knockdown plasmid of JUP (KD-JUP), or the negative control (NC) plasmids (OE-NC and KD-NC) (All provided by VectorBuilder Inc., Guangzhou, Guangdong, China). Each mouse was injected with 100 µL lentiviral solution through the tail vein at two weeks before ISO injection, and the virus titer was 8.8 × 10^12^ particles/mL. Based on the specific treatment and lentivirus injections, the mice were allocated into the sham, HCM, HCM + OE-NC, HCM + OE-Mycn, HCM + OE-NC, HCM + OE-USP2, HCM + OE-USP2 + KD-NC, and HCM + OE-USP2 + KD-JUP groups, *n* = 10 in each.

### Hematoxylin and eosin (HE) staining

Cardiac hypertrophy degree was assessed in heart tissue sections prepared from paraffined samples following the instruction of the HE staining kit (C0105M, Beyotime Biotechnology Co., Ltd., Shanghai, China). In short, the heart tissue sections were de-paraffined and rehydrated, followed by staining using hematoxylin solution at room temperature (22–25℃) for 10 min and eosin solution for 2 min. Subsequently, the sections were rehydrated in an ascending ethanol, made transparent in xylene, and mounted by neutral resin for microscopic observation.

### Echocardiography assessment

Before euthanasia, the mice were subjected to ultrasonic echocardiography measurements. An ultrasound probe with a frequency of 7.5 MHz was used. The left ventricular diameter during diastole and systole was measured from the short-axis view just beneath the mitral valve, to the right of the sternum. Data were collected over three cardiac cycles and averaged, and then the values of left ventricular ejection fraction (LVEF) and fractional shortening (LVFS) were calculated [16].

### Measurement of ROS

Generation of ROS in the mouse heart tissue was determined following the protocol of the ROS detection kit (DHE, dihydroethidium) (HR8821, Beijing Bio-Lab Technologies, Beijing, China). In brief, the tissue was made into homogenate and centrifuged to collect the supernatant. Subsequently, each well of 96-well plates was added with 200 µL supernatant and 2 µL DHE probe until fully mixed. The mixture was incubated at 37℃ in the dark for 15 min, and then the fluorescence intensity was determined using a fluorescence spectrometer.

### Terminal deoxynucleotidyl transferase (TdT)-mediated dUTP nick end labeling (TUNEL)

Cell apoptosis in the prepared heart tissue sections was determined using a TUNEL detection kit (C1098, Beyotime). In short, the biotin labeling solution, working solution, and chromogenic solution (DAB) were prepared following the kit’s protocol. The tissue sections were dewaxed, rehydrated, and dropped with DNase-free proteinase K (4333793, Thermo Fisher Scientific, Rockford, IL, USA) at 22–25℃. Subsequently, the sections were treated with the blocking reagent (SW3015, Solarbio Science & Technology Co., Ltd., Beijing, China), followed by incubation with 50 µL biotin labeling solution at 37℃ in the dark for 60 min, 0.3 mL termination reagent at 22–25℃ for 10 min, and 50 µL working solution at 22–25℃ for 30 min. Following this, 0.5 mL DAB solution was added for 30 min of color development. Subsequently, 50 µM 4’, 6-diamidino-2-phenylindole (DAPI) was added for 20 min of nuclear staining at 37℃. The sections were then dehydrated, cleared, and mounted for microscopic observation.

For mouse cardiomyocyte (see details in later text) labeling, the cells were fixed with 4% paraformaldehyde for 15 min and then penetrated using 0.5% Triton X-100 for 10 min. Subsequently, the cells were labeled with 50 µL TUNEL reagent (C1086, Beyotime) at 37℃ in the dark for 60 min, followed by washing and microscopic observation.

### Enzyme-linked immunosorbent assay (ELISA)

Blood sample of mice was collected from the heart valves using cardiac puncture and centrifuged at 2000 *g* at 4 °C for 10 min. The concentrations of creatine kinase (CK), CK-MB, lactate dehydrogenase (LDH), and cardiac troponin T (cTnT) in the mouse serum, and the concentrations of superoxide dismutase (SOD), malondialdehyde (MDA), atrial natriuretic peptide (ANP), and brain natriuretic peptide (BNP) in the mouse heart tissues were analyzed following the instruction manuals of the mouse CK (E-BC-K558-S, Elabscience Biotechnology Co., Ltd., Wuhan, Hubei, China), CK-MB (D721065, Sangon Biotech (Shanghai) Co., Ltd., Shanghai, China), LDH (A020-1-1, JianCheng Bioengineering Institute, Jiangsu, China), cTnT/TNNT2 (D721161, Sangon), SOD (D799594, Sangon), MDA (D799761, Sangon), ANP (69-20098, MSK Biotechnologies, Wuhan, Hubei, China), and BNP (D721185, Sangon) ELISA kits. All detection was performed in strict accordance with the manufacturers’ instructions.

### Reverse transcription-quantitative polymerase chain reaction (RT-qPCR)

To analyze the mRNA expression of Mycn, USP2, and JUP, the mouse heart tissues and cardiomyocytes were collected, in which the total RNA sample was isolated using the TRIzol reagent. The RNA reverse transcription and real-time qPCR analysis was performed using the One Step TB Green® PrimeScript™ RT-PCR Kit (RR066A, Takara Holdings Inc., Kyoto, Japan) and the CFX96 Touch™ real-time qPCR system (Bio-Rad, Inc., Hercules, CA, USA). The gene expression was determined using the 2^−ΔΔCt^ method with β-actin used as the internal control. Following are the primer sequences: Mycn (F) 5ʹ-TGTGTCTGTGTTCCAGCISOTGCC-3ʹ, Mycn (R) 5ʹ-CATCTTCCTCCTCGTCATCCTC-3ʹ; USP2 (F) 5ʹ-AGCCCATCTGAGTTCAAGACCC-3ʹ, USP2 (R) 5ʹ-GGTTCACCTCATTGTGGAGACC-3ʹ; JUP (F) 5ʹ-ACCAGCATCCTGCAACCTCT-3ʹ, JUP (R) 5ʹ-CAGTGTGGTGATGGCGTAGAAC-3ʹ; β-MHC (F) 5ʹ-GCTGGAAGATGAGTGCTCAGAG-3ʹ, β-MHC (R) 5ʹ-TCCAAACCAGCCATCTCCTCTG-3ʹ; ANP (F) 5ʹ-GATCTCAGCACAATAGAGCCGC-3ʹ, ANP (R) 5ʹ-CCTGTCATAGCCATCGAGGTAC-3ʹ; BNP (F) 5ʹ-TCCTAGCCAGTCTCCAGAGCAA-3ʹ, BNP (R) 5ʹ-GGTCCTTCAAGAGCTGTCTCTG-3ʹ; β-actin (F) 5ʹ-CATTGCTGACAGGATGCAGAAGG-3ʹ, β-actin (R) 5ʹ-TGCTGGAAGGTGGACAGTGAGG-3ʹ.

### Cells and treatment

Mouse cardiomyocytes (CP-M073, Procell Life Science & Technology Co., Ltd., Wuhan, Hubei, China) were cultured in the specific complete medium (CM-M073) at 37℃ with 5% CO_2_. Gene overexpression plasmids of Mycn and JUP2 (OE-Mycn and OE-JUNP) and the OE-NC were transfected into the mouse cardiomyocytes using Lipofectamine 3000 (L3000075, Thermo Fisher Scientific). After that, the cells were cultured at 37℃ with 5% CO_2_ for 48 h and then transferred into fresh medium. After successful transfection, the cells were co-cultured with 20 µM ISO for 48 h to induce hypertrophic growth of cardiomyocytes in vitro. Cells cultured with phosphate-buffered saline (PBS) were set to controls.

### Chromatin immunoprecipitation (ChIP) assay

The binding between Mycn and the USP2 promoter was examined using the ChIP assay kit (P2078, Beyotime). Briefly, the cells were crosslinked in 1% formaldehyde for 10 min and ultrasonicated on ice for chromatin truncation. The cell lysates were reacted with the antibodies of Mycn (1:100, ab16898, Abcam Inc., Cambridge, MA, USA) or IgG (1:100, sc-2025, Santa Cruz Biotechnology, Inc, Santa Cruz, CA, USA). The precipitated immune complexes were collected using agarose magnetic beads, isolated, and de-crosslinked using NaCl at 65℃. The DNA was then extracted and purified using the phenol-chloroform-isoamyl alcohol system, in which the abundance of USP2 promoter fragments was analyzed using qPCR.

### Luciferase assay

The promoter sequence of USP2 was queried in the UCSC system (http://genome.ucsc.edu/index.html), which was amplified and cloned into the pGL6 luciferase reporter vectors (D2102, Beyotime). The reporter vector was co-transfected with OE-Mycn into the mouse cardiomyocytes using Lipofectamine 3000. After 48 h, the cells were collected and lysed, in which the luciferase activity was analyzed utilizing a dual luciferase reporter system.

### Ubiquitination and co-immunoprecipitation (co-IP) assay

Mouse cardiomyocytes were treated with 10 µM MG132 (S1748, Beyotime) for 24 h and lysed in radio-immunoprecipitation assay (RIPA) lysis buffer with the supernatant collected. Co-IP reaction was then performed using the JUP antibody (1:1000, PA5-29930, Thermo Fisher Scientific). The immunoprecipitants were collected using magnetic beads and isolated, and the levels of Ub (1:500, 48860, Signalway Antibody LLC, Greenbelt, MA, USA), JUP, and USP2 (1:1000, PA5-48091, Thermo Fisher Scientific) were analyzed using western blot (WB) analysis.

### Cycloheximide (CHX) treatment

CHX (SR0222C, Thermo Fisher Scientific), a protein synthesis inhibitor, was used to examine the protein stability in cells. Briefly, the OE-USP2-transfected mouse cardiomyocytes were treated with 50 mg/mL CHX. After 0 h, 2 h, 4 h, 6 h, and 8 h, the protein level of JUP in cells was analyzed using WB analysis.

### WB analysis

Total protein from mouse heart tissues or cardiomyocytes was extracted using the RIPA lysis buffer. After protein concentration analysis using the bicinchoninic acid (BCA) kit, the protein was denaturalized in water bath and separated by 10% sodium dodecyl sulfate-polyacrylamide gel electrophoresis to transfer onto polyvinylidene fluoride membranes. The membranes were blocked with 5% goat serum for 1 h and incubated with the antibodies of B-cell lymphoma-2 (Bcl-2; 1:1000, GTX100064, GeneTex Inc., San Antonio, TX, USA), Bcl-2-associated X (Bax; 1:1500, GTX109683, GeneTex), cleaved-caspase-3 (c-CAS-3, 1:5000, ab214430, Abcam), interleukin-1β (IL-1β; 1:1500, GTX74034, GeneTex), matrix metalloproteinase-9 (MMP-9; 1:1000, ab283575, Abcam), tumor necrosis factor-α (TNF-α; 1:1000, 3707S, Cell Signaling Technology, Beverly, MA, USA), USP2 (1:1000, PA5-48091, Thermo Fisher Scientific), JUP (1:1000, PA5-29930, Thermo Fisher Scientific), pAKT (1:500, AF5734, Beyotime), β-catenin (1:5000, GTX101435, GeneTex), and GAPDH (1:5000, 48142, Signalway Antibody LLC) overnight at 4℃. On the next day, the membranes were incubated with HRP-conjugated goat anti-rabbit IgG (1:2000, #7074, Cell Signaling Technology) or rabbit anti-goat IgG (1:2000, D110121, Sangon) at 22–25℃ for 1 h. The blot bands were developed using enhanced chemiluminescence analyzed using Image J. GAPDH was used as the endogenous control for protein quantification.

### Cell viability detection

Viability of mouse cardiomyocytes was analyzed using the 3-(4, 5-dimethylthiazol-2-yl)-2, 5-diphenyltetrazolium bromide (MTT) assay. Following the instructions of the MTT kit (C0009S, Beyotime), the MTT solution at 5 mg/mL was prepared, and each well was added with 5,000 cells (100 µL), followed by the addition of 10 µL MTT solution for 4 h of incubation. Subsequently, each well was added with 100 µL formazan solvent for 4 h. The optical density at 570 nm was examined to evaluate cell viability.

### Data analysis

All experimental data were collected from at least three biological replicates and analyzed using Prism 8.0.2 (GraphPad, La Jolla, CA, USA). The data are presented in the form of mean ± standard deviation. Differences between groups were determined using unpaired *t* test, one-way analysis of variance (ANOVA), or two-way ANOVA, as appropriate. Tukey’s multiple comparison was used for the post-hoc examination after ANOVA. Statistically significant differences were defined by *p* < 0.05.

## Results

### Mycn is poorly expressed in mice with hypertrophic cardiomyopathy

**Fig. 1:**
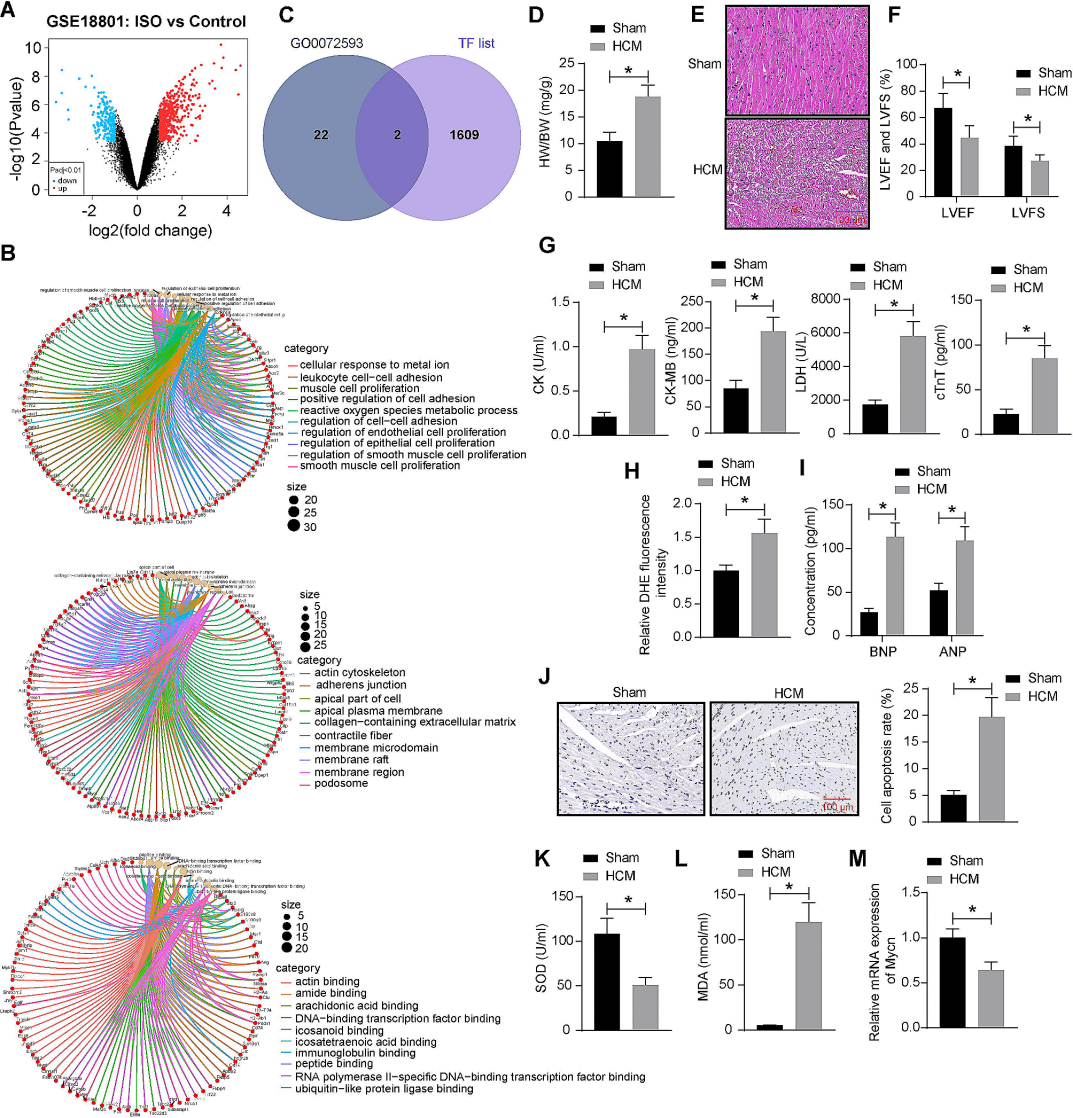
Mycn is poorly expressed in mice with cardiac hypertrophy. **A**, DEGs (|Log2Foldchange| > 1 and *p* < 0.01) between ISO-induced hearts and hearts of healthy control mice analyzed using the GEO dataset GSE18801. **B**, A GO enrichment analysis of the DEGs. **C**, a Venn diagram for the intersecting genes between DEGs and mouse transcription factors predicted using the AnimalTFDB v4.0 system. Mice were subcutaneously injected with ISO or PBS. **D**, HW/BW of mice. **E**, Myocardial injury of mice analyzed by HE staining. **F**, LVEF and LVFS or mice analyzed by echocardiographic assessment. **G**, Serum levels of CK, CK-MB, LDH, and cTnT in mice determined using ELISA kits. **H**, ROS content in the mouse heart determined using DHE staining. **I**, Concentrations of ANP and BNP in the mouse heart tissue determined using ELISA kits. **J**, Cell apoptosis in the mouse heart tissue examined using TUNEL assay. **K**-**L**, SOD activity (K) and MDA content (L) in the mouse heart tissue determined using ELISA kits. **M**, Mycn mRNA expression in the mouse heart tissue examined by RT-qPCR. In each group, *n* = 10. Differences were analyzed using the unpaired *t* test or the two-way ANOVA. *p < 0.05

Differentially expressed genes (DEGs) between ISO-induced hearts and hearts of healthy control mice were analyzed using the GEO dataset GSE18801 (https://www.ncbi.nlm.nih.gov/geo/query/acc.cgi?acc=GSE18801). The selection criteria for significance were set at |Log2Foldchange| > 1 and *p* < 0.01 (Fig. 1A). Subsequently, a gene ontology (GO) enrichment analysis of the DEGs revealed their involvement in various cellular component terms, such as actin cytoskeleton, apical plasma membrane, and collagen-containing extracellular matrix. Additionally, the DEGs participated in biological processes like ROS metabolic process and muscle cell proliferation, and exhibited molecular functions such as DNA-binding transcription factor binding and RNA polymerase II-specific DNA-binding transcription factor binding (Fig. 1B). Given the close association between oxidative stress and the progression of hypertrophic cardiomyopathy, genes enriched in the “GO0072593: ROS metabolic process” pathway were selected for subsequent analyses. These genes were cross-referenced with the transcription factors (Mus musculus) from AnimalTFDB v4.0 (http://bioinfo.life.hust.edu.cn/AnimalTFDB4/#/Download), yielding two intersections: Foxo3 and Mycn (Fig. 1C). We chose Mycn for further analysis. In the GSE18801 dataset, Mycn exhibits a low-expression pattern (Log2Foldchange = -1.02316491, *p* = 0.00359573) in ISO-induced hearts. Subsequently, a mouse model of cardiac hypertrophy was generated by ISO injection. After 9 d, the ISO-treated mice showed significantly increased HW/BW (Fig. 1D). Additionally, the HE staining showed disorganized myocardial cells, increased cell surface area, inflammatory cell infiltration, interstitial congestion, and disrupted myocardial fiber alignment in the heart tissue of ISO-treated mice (Fig. 1E). Echocardiographic assessment showed that the ISO-treated mice had a significant reduction in LVEF and a significant decrease in LVFS compared to the sham group (Fig. 1F). Furthermore, blood sample analyses showed that the model mice had significantly elevated serum levels of CK, CK-MB, LDH, and cTnT compared to the sham-operated mice (Fig. 1G). The DHE fluorescence staining showed that the ROS content was increased in the ISO-treated mice as well (Fig. 1H). In addition, the modeled mice had increased concentrations of ANP and BNP (Fig. 1I), increased number of TUNEL-positive cells (Fig. 1J), and increased content of MDA while decreased activity of SOD (Fig. 1K-L) in their heart tissue, indicating the heart dysfunction, increased cardiomyocyte apoptosis, and oxidative stress. This evidence proves the successful modeling of HCM. Notably, RT-qPCR showed that the mRNA expression of Mycn was conspicuously increased in the heart of HCM mice (Fig. 1M).

### Mycn upregulation alleviates myocardial dysfunction in mice

**Fig. 2:**
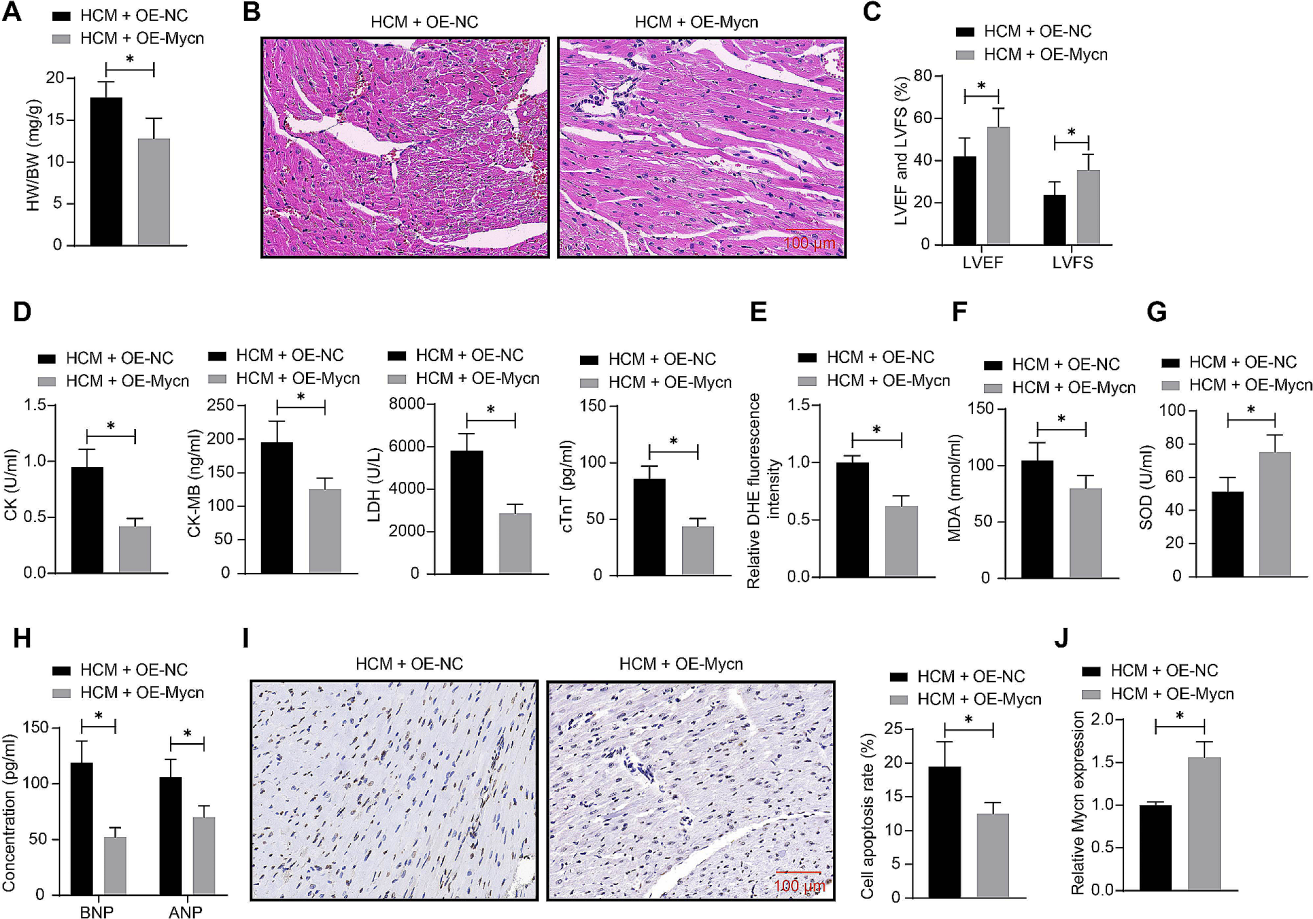
Mycn upregulation alleviates myocardial dysfunction in mice. Mice were administrated with lentiviral vectors-carried OE-Mycn or OE-NC, followed by ISO injection. **A**, HW/BW of mice. **B**, Myocardial injury of mice analyzed by HE staining. **C**, LVEF and LVFS or mice analyzed by echocardiographic assessment. **D**, Serum levels of CK, CK-MB, LDH, and cTnT in mice determined using ELISA kits. **E**, ROS content in the mouse heart determined using DHE staining. **F**-**G**, MDA content (F) and SOD activity (G) in the mouse heart tissue determined using ELISA kits. **H**, Concentrations of ANP and BNP in the mouse heart tissue determined using ELISA kits. **I**, Cell apoptosis in the mouse heart tissue examined using TUNEL assay. **J**, Mycn mRNA expression in the mouse heart tissue examined by RT-qPCR. In each group, *n* = 10. Differences were analyzed using the unpaired *t* test or the two-way ANOVA. *p < 0.05

To assess the biological activity of Mycn in cardiac hypertrophy-induced heart failure, upregulation of Mycn was induced in mice using lentiviral vector-carried OE-Mycn, followed by ISO injection. Compared to OE-NC, the OE-Mycn administration significantly decreased the HW/BW of mice (Fig. 2A). The OE-Mycn administration also improved the arrangement of cardiomyocytes and myocardial fibers while decreasing cell surface area, inflammatory cell infiltration, and interstitial congestion in the heart of the HCM mice (Fig. 2B). Meanwhile, it increased the LVEF and LVFS of mice (Fig. 2C). The biochemical analyses further revealed that the OE-Mycn administration decreased the serum levels of CK, CK-MB, LDH, and cTnT (Fig. 2D), reduced the cardiac ROS content (Fig. 2E), increased SOD activity while decreasing MDA content in the heart (Fig. 2F-G), reduced the cardiac ANP and BNP levels (Fig. 2H), and decreased the number of TUNEL-positive cells in the heart (Fig. 2I). Indeed, RT-qPCR results showed that the Mycn expression in the heart tissue of mice was successfully increased by OE-Mycn (Fig. 2J).

### Mycn possesses cardiomyocyte-protective effects in vitro

**Fig. 3:**
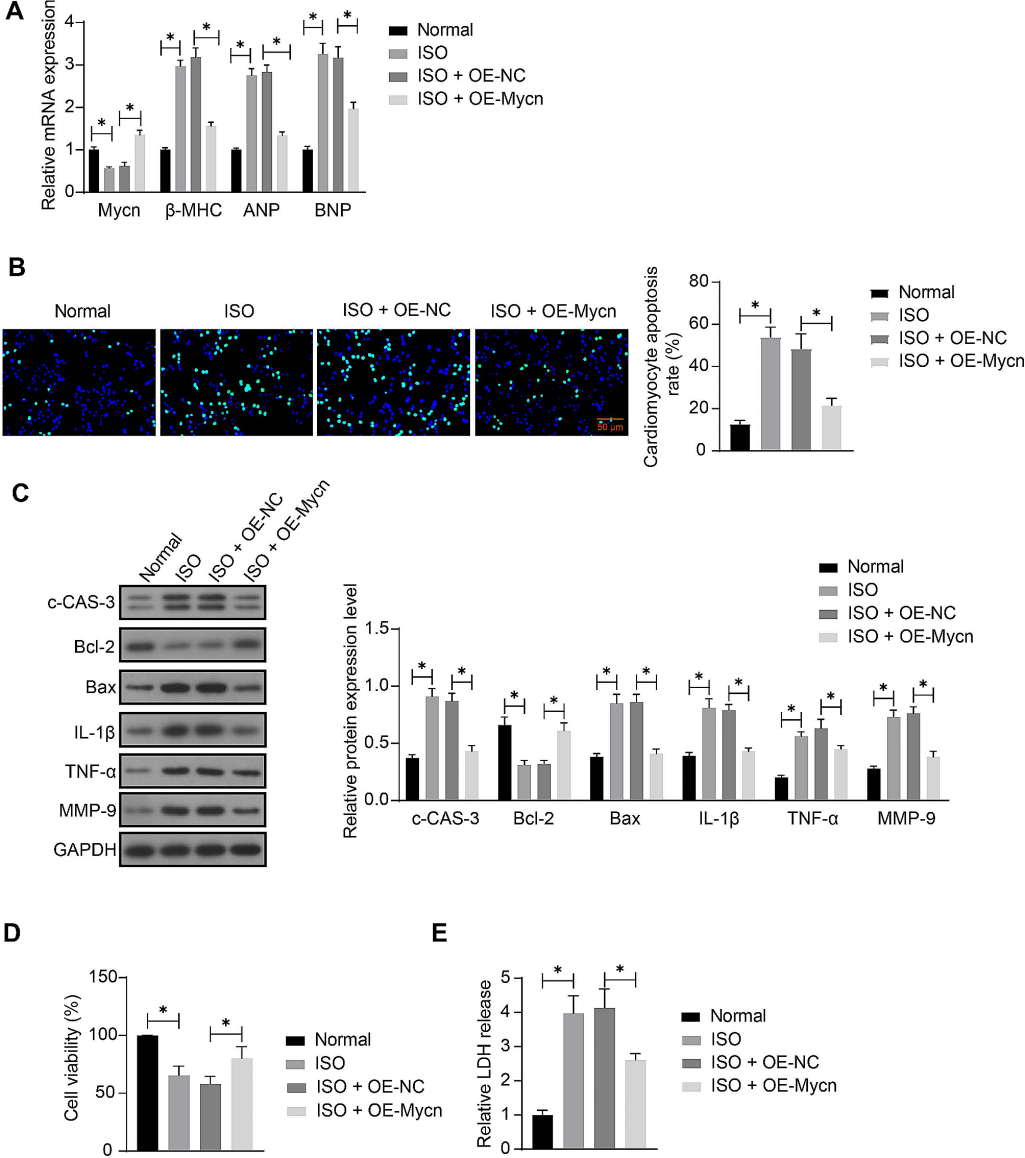
Mycn possesses cardiomyocyte-protective effects in vitro. **A**, mRNA expression of Mycn, ANP, BNP, and β-MHC in cardiomyocytes after OE-Mycn/OE-NC transfection and ISO treatment examined by RT-qPCR; **B**, Apoptosis of cardiomyocytes examined by TUNEL assay; **C**, Protein levels of c-CAS-3, Bcl-2, Bax, IL-1β, TNF-α, and MMP-9 in cardiomyocytes determined using WB analysis; **D**, Viability of cardiomyocytes examined using MTT assay; **E**, LDH release in cardiomyocytes analyzed using ELISA. Three biological replicates were performed. Differences were analyzed using the one-way or two-way ANOVA. *p < 0.05. See full-length blot images in Supplementary Figure S1-S2

Mouse cardiomyocytes, either with or without OE-Mycn/OE-NC transfection, were treated with ISO to induce hypertrophic growth. The ISO significantly decreased the Mycn expression, and the administration of OE-Mycn successfully increased the Mycn (Fig. 3A). In addition, it was found that the mRNA expression of the hypertrophy markers ANP, BNP, and β-MHC in cells was increased by ISO treatment but decreased after Mycn upregulation (Fig. 3A). The TUNEL assay showed that the Mycn overexpression protected the cardiomyocytes from ISO-induced apoptosis (Fig. 3B). Consistently, the WB analysis showed that the protein levels of apoptosis-related markers c-CAS-3 and Bax and the inflammatory factors IL-1β, TNF-α, and MMP-9 in the cardiomyocytes were increased by ISO but decreased by OE-Mycn, while the anti-apoptotic protein Bcl-2 was decreased by ISO but rescued by OE-Mycn (Fig. 3C) (Supplementary Fig S1-S2). MTT assay showed that the viability of cardiomyocytes was decreased by ISO but maintained upon Mycn overexpression (Fig. 3D). By contrast, the LDH release in the cardiomyocytes was promoted by ISO treatment but decreased under Mycn overexpression (Fig. 3E).

### Mycn activates USP2 transcription

**Fig. 4:**
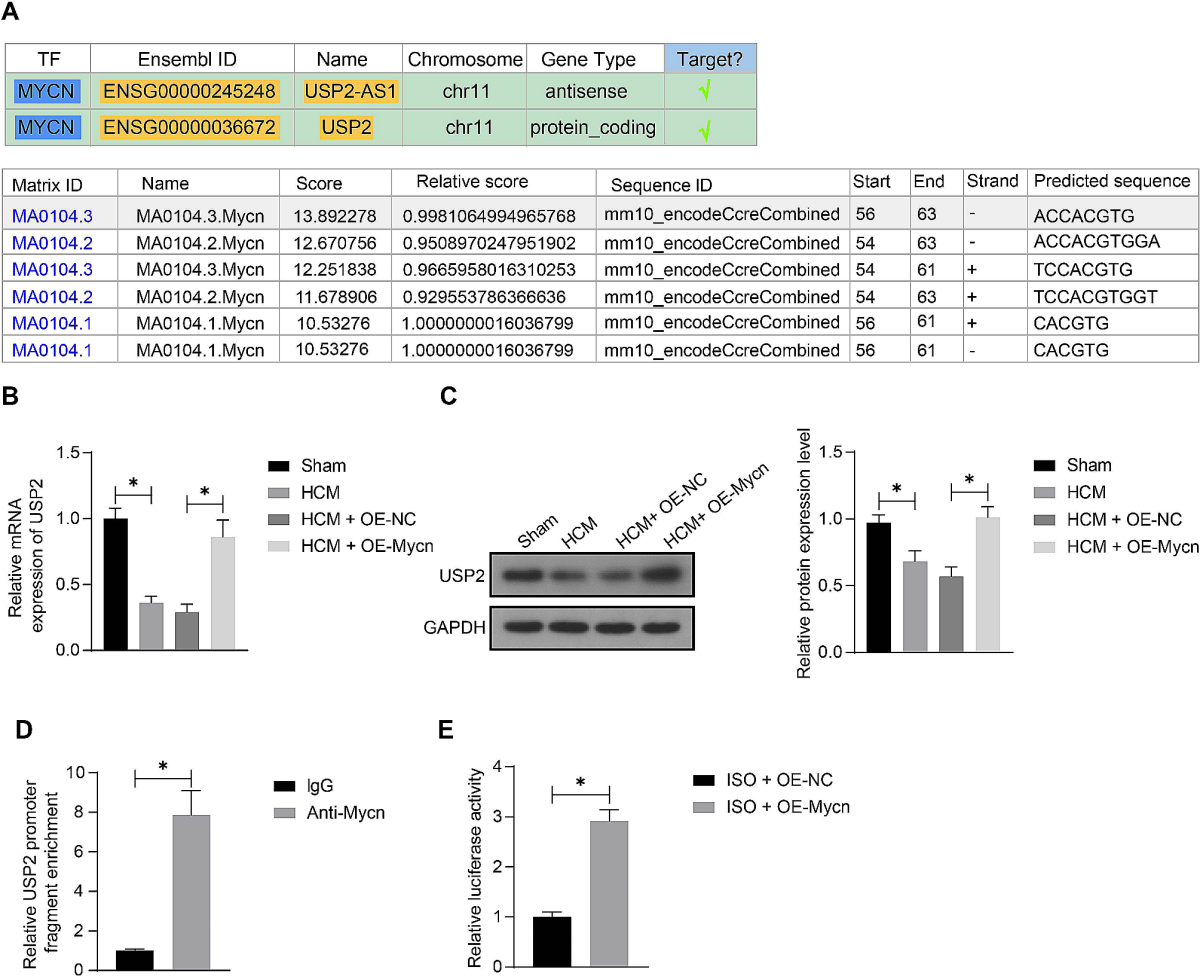
Mycn activates USP2 transcription. **A**, USP2 predicted as a target of Mycn predicted in the hTFtarget system and the putative binding sites. **B**-**C**, mRNA (B) and protein (C) expression of USP2 in the heart tissue of mice after HCM modeling and OE-Mycn treatment examined by RT-qPCR and WB analysis, respectively. **D**, binding relationship between Mycn and the USP2 promoter in mouse cardiomyocytes determined by the ChIP assay. **E**, transcription activity of USP2 promoter in mouse cardiomyocytes transfected with OE-Mycn examined by the luciferase reporter gene assay. For animal experiments, *n* = 10 in each group. For cellular experiments, three biological replicates were conducted. Differences were analyzed by the unpaired *t* test or the one-way ANOVA. *p < 0.05. See full-length blot images in Supplementary Figure S3

To probe the possible mechanism involved in the protective events of Mycn, we predicted the downstream targets of Mycn, as a transcription factor, in the hTFtarget system (http://bioinfo.life.hust.edu.cn/hTFtarget/#!/). Among the possible targets, USP2 attracted our interests (Fig. 4A). In addition, according to data in the JASPAR system (https://jaspar.genereg.net/), highly reliable binding sites of Mycn on the USP2 promoter were predicted. It is inferred that the beneficial effect of Mycn on cardiac hypertrophy depends on the transcriptional activation of USP2. Importantly, RT-qPCR showed that the USP2 mRNA expression in the heart tissue was decreased in the ISO-treated mice compared to the sham-operated ones, and this decrease was blocked following Mycn overexpression (Fig. 4B). The protein level of USP2 in the heart tissue presented a similar trend (Fig. 4C) (Supplementary Fig S3). In addition, in the ChIP-qPCR assay, we found an enrichment of USP2 promoter fragments in the immunoprecipitants reacted with the Mycn antibody (Fig. 4D). The luciferase assay showed that the Mycn overexpression in mouse cardiomyocytes significantly increased the luciferase activity of the reporter vector containing the USP2 promoter sequence (Fig. 4E). Collectively, this evidence suggests that the Mycn binds to the USP2 promoter for its transcription activation.

### USP2 overexpression alleviates cardiac hypertrophy and dysfunction in mice

**Fig. 5:**
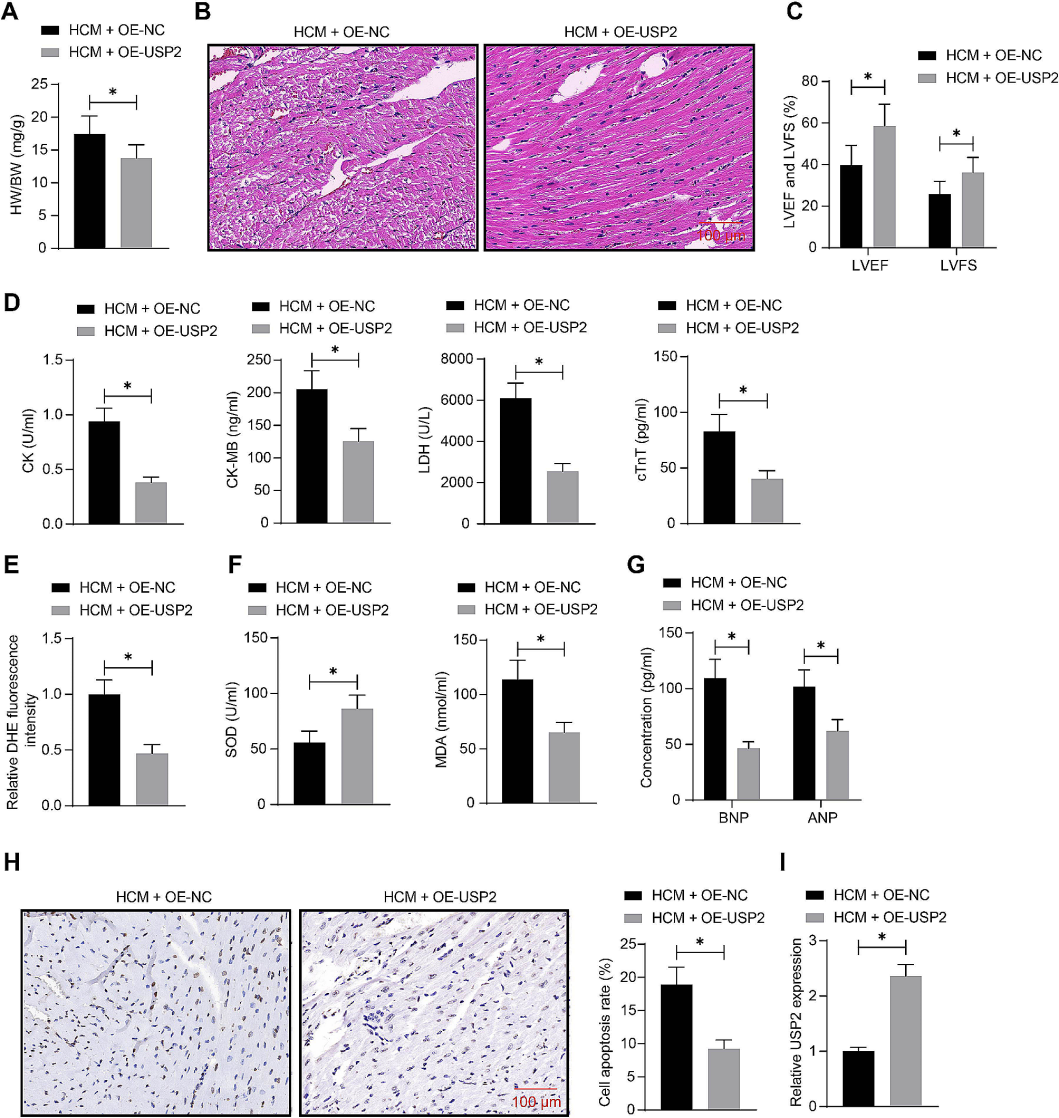
USP2 overexpression alleviates cardiac hypertrophy and dysfunction in mice. Mice were administrated with lentiviral vectors-carried OE-USP2 or OE-NC, followed by ISO injection. **A**, HW/BW of mice. **B**, Myocardial injury of mice analyzed by HE staining. **C**, LVEF and LVFS or mice analyzed by echocardiographic assessment. **D**, Serum levels of CK, CK-MB, LDH, and cTnT in mice determined using ELISA kits. **E**, ROS content in the mouse heart determined using DHE staining. **F**, SOD activity and MDA content in the mouse heart tissue determined using ELISA kits. **G**, Concentrations of ANP and BNP in the mouse heart tissue determined using ELISA kits. **H**, Cell apoptosis in the mouse heart tissue examined using TUNEL assay. **I**, Mycn mRNA expression in the mouse heart tissue examined by RT-qPCR. In each group, *n* = 10. Differences were analyzed using the unpaired *t* test or the two-way ANOVA. *p < 0.05

Lentiviral vector-carried OE-USP2 was administrated into mice, followed by ISO injection, to analyze the function of USP2 in cardiac hypertrophy. Like OE-Mycn, the OE-USP2 administration also decreased the HW/BW of mice (Fig. 5A). It also alleviated pathological changes and reduced cell surface area in the heart of the ISO-treated mice (Fig. 5B). OE-USP2 also increased the LVEF and LVFS of mice (Fig. 5C), and it decreased the serum levels of CK, CK-MB, LDH, and cTnT (Fig. 5D), reduced the cardiac ROS content (Fig. 5E), and increased SOD activity while reducing MDA concentration in the mouse heart (Fig. 5F). Furthermore, the OE-USP2 administration decreased the cardiac ANP and BNP levels (Fig. 5G) and decreased cell apoptosis in the mouse heart (Fig. 5H). In addition, successful upregulation of USP2 was detected in the mouse heart after OE-USP2 injection (Fig. 5I).

### USP2 stabilizes JUP protein to block the Akt/β-catenin signaling cascade

**Fig. 6:**
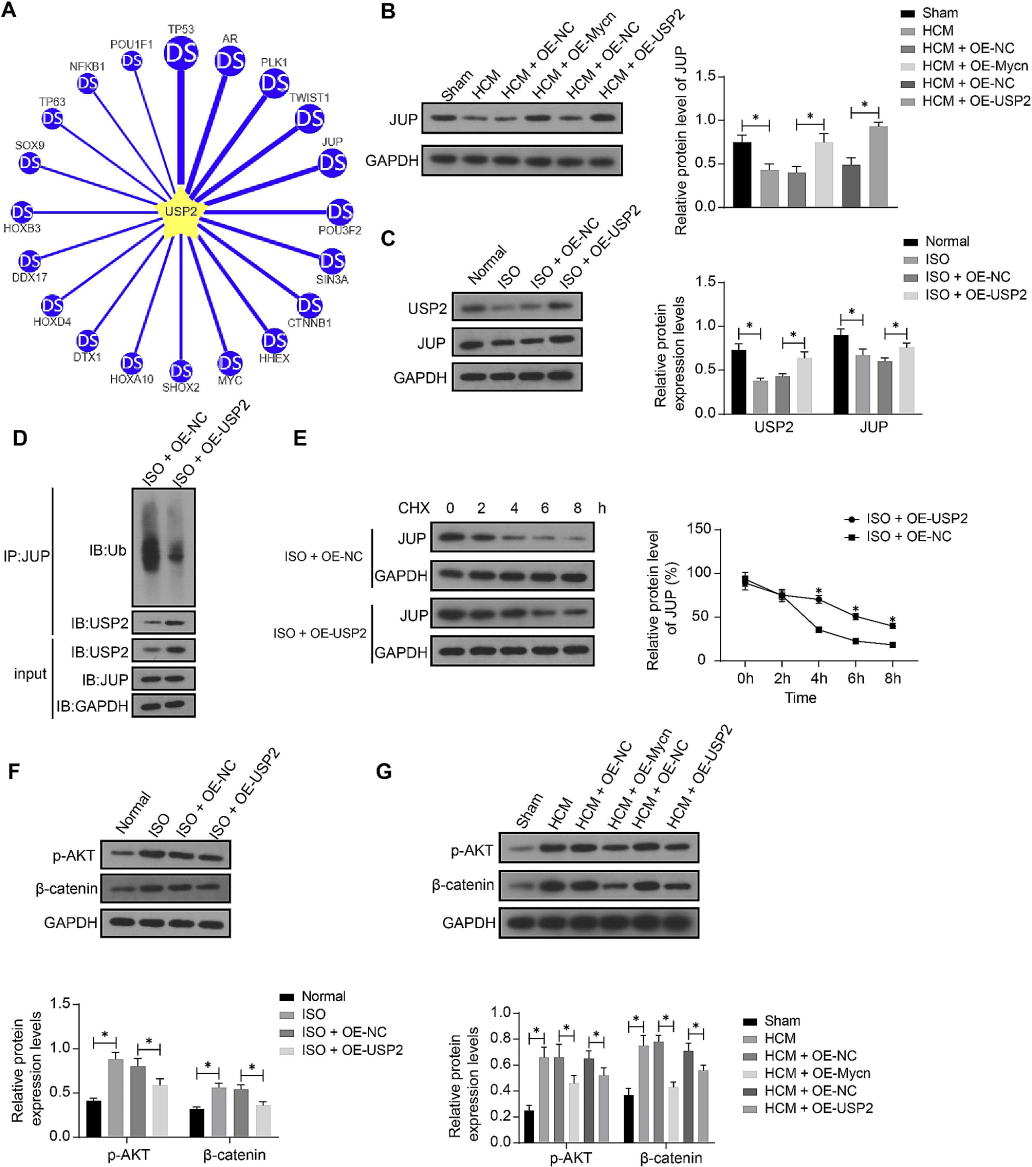
USP2 stabilizes JUP protein to block the Akt/β-catenin signaling cascade. **A**, Possible substrates of USP2 predicted in the UbiBrowser system. **B**, JUP protein level in mouse heart tissues examined by WB analysis. See full-length blot images in Supplementary Figure S4. **C**, protein levels of USP2 and JUP in mouse cardiomyocytes after OE-USP2 transfection determined by WB analysis. See full-length blot images in Supplementary Figure S5. **D**, ubiquitination of JUP mediated by USP2 examined by ubiquitination and co-IP assays. Supplementary Figure S6-S7. **E**, JUP protein degradation rates in mouse cardiomyocytes under CHX treatment. Supplementary Figure S8-S9. **F**, phosphorylation of Akt and protein level of β-catenin in the mouse cardiomyocytes determined by WB analysis. Supplementary Figure S10. **G**, phosphorylation of Akt and protein level of β-catenin in mouse heart tissue examined by WB analysis. Supplementary Figure S11. For animal experiments, *n* = 10 in each group. For cellular experiments, three biological replicates were conducted. Differences were analyzed by the unpaired *t* test or the two-way ANOVA. *p < 0.05

To investigate the downstream molecular mechanism of the deubiquitinase USP2, we predicted the substrates of USP2 using UbiBrowser 2.0 (http://ubibrowser.bio-it.cn/ubibrowser_v3/) (Fig. 6A). JUP was identified as an interesting target worthy further investigation considering its regulation on the Akt/β-catenin cascade mentioned in the earlier text. We hypothesized that USP2 possibly mediates JUP deubiquitination and inactivates the Akt/β-catenin cascade to ameliorate cardiac hypertrophy. Importantly, decreased JUP expression was detected in the ISO-treated mice, which was restored by either OE-Mycn or OE-USP2 (Fig. 6B) (Supplementary Fig S4). OE-USP2 was transfected into the mouse cardiomyocytes, followed by ISO treatment. The OE-USP2 transfection successfully increased the USP2 expression in the cells, which also led to an increase in the JUP protein level (Fig. 6C) (Supplementary Fig S5). Subsequently, the ubiquitination and co-IP assay confirmed that USP2 modified the deubiquitination of JUP protein (Fig. 6D) (Supplementary Fig S6-S7). Under CHX treatment, the USP2 overexpression decreased the degradation rate of JUP protein in the cardiomyocytes (Fig. 6E) (Supplementary Fig S8-S9). When it comes to the Akt/β-catenin signaling activity, it was found that the phosphorylation of Akt and protein level of β-catenin in the mouse cardiomyocytes were promoted after ISO treatment but decreased after USP2 overexpression (Fig. 6F) (Supplementary Fig S10). In vivo, likewise, increased Akt phosphorylation and β-catenin protein level were detected in the mouse heart after ISO treatment, which were blocked by OE-Mycn or OE-USP2 as well (Fig. 6G) (Supplementary Fig S11).

### Knockdown of JUP activates the Akt/β-catenin signaling and aggravates cardiac impairment

**Fig. 7:**
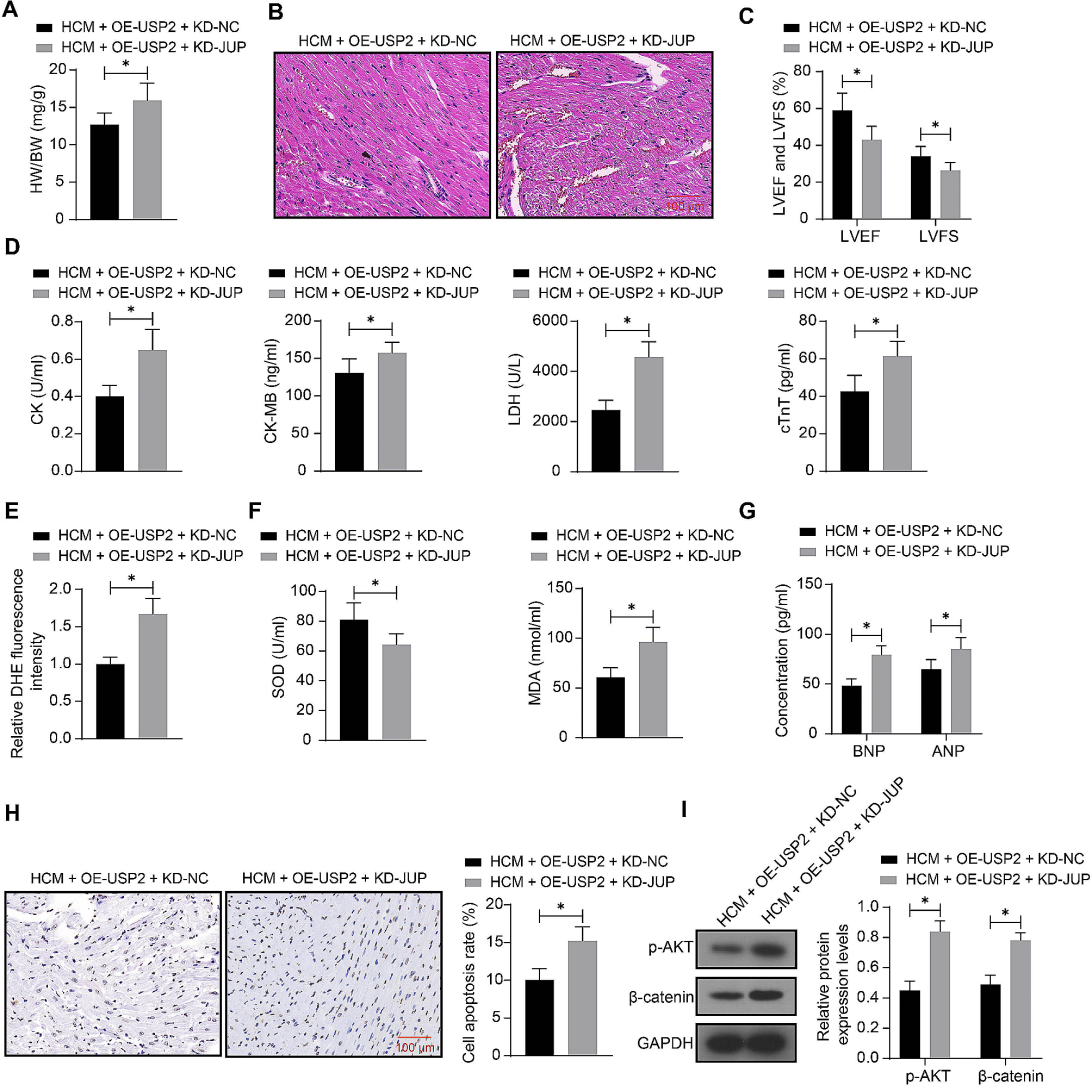
Knockdown of JUP activates the Akt/β-catenin signaling and aggravates cardiac impairment. Mice were administrated with lentiviral vectors-carried OE-USP2 and the additional KD-JUP or KD-NC, followed by ISO injection. **A**, HW/BW of mice. **B**, Myocardial injury of mice analyzed by HE staining. **C**, LVEF and LVFS or mice analyzed by echocardiographic assessment. **D**, Serum levels of CK, CK-MB, LDH, and cTnT in mice determined using ELISA kits. **E**, ROS content in the mouse heart determined using DHE staining. **F**, SOD activity and MDA content in the mouse heart tissue determined using ELISA kits. **G**, Concentrations of ANP and BNP in the mouse heart tissue determined using ELISA kits. **H**, Cell apoptosis in the mouse heart tissue examined using TUNEL assay. **I**, phosphorylation of Akt and protein level of β-catenin in mouse heart tissue examined by WB analysis. In each group, *n* = 10. Differences were analyzed using the unpaired *t* test or the two-way ANOVA. *p < 0.05. See full-length blot images in Supplementary Figure S12

To validate if the JUP upregulation is involved in the myocardium-protective events of USP2, the mice administrated with OE-USP2 were further administrated with KD-JUP, followed by ISO treatment. Importantly, it was found that the KD-JUP restored the HW/BW (Fig. 7A), increased the cell surface area and inflammatory infiltration in the heart tissue (Fig. 7B), decreased LVEF and LVFS (Fig. 7C), and increased the serum levels of CK, CK-MB, LDH, and cTnT (Fig. 7D). The KD-JUP also increased the ROS content (Fig. 7E), reduced SOD activity and increased MDA content (Fig. 7F), increased the concentrations of ANP and BNP (Fig. 7G), and increased cell apoptosis (Fig. 7H) in the mouse heart tissue. Importantly, the KD-JUP was found to restore the Akt phosphorylation and β-catenin protein level in the mouse heart tissue (Fig. 7I) (Supplementary Fig S12).

## Discussion

Pathological cardiac hypertrophy represents a significant risk element for heart failure and potentially even abrupt fatality [17, 18]. Handling this cardiovascular disease continues to pose difficulties within clinical settings. In this study, we found that aberrant low Mycn expression is implicated in the pathogenesis of cardiac failure. Restoration of Mycn was found to alleviate hypertrophic growth, oxidative stress, and apoptosis of cardiomyocytes both in vitro and in vivo.

First, a mouse model of HCM was induced by ISO treatment. The mice presented significantly increased HW/BW, hypertrophic growth and disarray of cardiomyocytes, inflammation infiltration, and oxidative stress and cell apoptosis, which are typical histological characteristics of HCM [19]. Bioinformatics analyses in this work showed that the DEGs in the GSE18801 microarray dataset are enriched in several biological processes, including the ROS metabolic process. Indeed, oxidative stress has been identified as a key inducer of both genetic and acquired cardiac hypertrophy, and patients with HCM usually present increased levels of circulating oxidative markers [20]. In fact, increased oxidative stress is also a primary mechanism for cardiomyocyte apoptosis following cardiac hypertrophy [21]. Furthermore, we obtained Mycn and Foxo3 as two promising mediators of oxidative stress that are deregulated during HCM. Studies concerning the function of Foxo3 in HCM abound. For instance, Foxo3-mediated antioxidative signaling pathway has been demonstrated to be responsible for the anti-hypertrophic events following CD38 gene silencing [22]. Similarly, silencing of Foxo3 has been associated with hypertrophic growth of cardiomyocytes [23], and vice versa [24]. Concerning Mycn, it has a close correlation with the progression of neuroblastoma [18, 25]. Mutations in the Mycn gene has been correlated with Feingold syndrome, a developmental disorder characterized in part by congenital heart defects [26]. The Mycn deletion in mouse myocardium has also been associated with myocardial wall thickening [8]. However, the functions of Mycn in heart protection in the context of HCM remains largely unknown and interesting. Of note, we found that the Mycn upregulation reduced hypertrophic growth and disarray of cardiomyocytes, inflammation, oxidative stress, and apoptosis in HCM mice. Indeed, the Mycn amplification in neuroblastoma cells has been correlated with resistance to oxidative stress [27]. This also implicates a potential inverse correlation between Mycn expression and oxidative stress activity in cells. Moreover, we also found that the Mycn reduced ISO-induced apoptosis of cardiomyocytes and the expression of ANP, BNP and β-MHC, which are hypertrophy-related biomarkers that are frequently related to maladaptive cardiac remodeling and dysfunction [6]. However, more detailed mechanism involved in the anti-hypertrophic effects of Mycn require further investigation.

As a bHLH transcription factor, Mycn controls transcription networks that orchestrate cell growth, differentiation, and death [28]. Notably, we obtained USP2 as a promising target with an existence of highly reliable binding sites of Mycn on its promoter. Overexpression of USP2 has been found to protect the heart from pressure overload-induced cardiac remodeling, cardiac hypertrophy, inflammation, and oxidative stress in a mouse model induced by transverse aortic constriction [29]. Likewise, in a recent study by Fu et al., USP2 overexpression has been found to reduce cell surface area, alleviate mitochondrial dysfunction and oxidative stress, and decrease the ANP, BNP and β-MHC levels both in animal and cellular models of hypertrophic growth induced by Angiotensin II [26]. Here, we observed that the USP2 expression was decreased in mice with HCM and restored by Mycn overexpression. Indeed, the USP2 upregulation exerted myocardium-protective effects like Mycn.

Regarding the downstream substrates involved in the events mediated by USP2, we obtained by bioinformatics that TP53, AR, PLK1, TWIST1, and JUP are the top 5 most relevant targets of USP2. Considering that the exact roles of TP53 [30], AR [31], PLK1 [32], and TWIST1 [33] in myocardial hypertrophy have been investigated, we chose JUP for further investigation. Indeed, we found that the USP2 overexpression in cardiomyocytes enhanced the protein stability of JUP, which is a novel interaction that has been reported before. JUP is a crucial desmosomal protein in the heart whose dysfunction may lead to cardiomyocyte detachment, signaling transduction, and cardiac fibrosis and arrhythmias [34]. Indeed, as introduced earlier, cardio-restricted knockout of USP2 in mice led to activation of the Akt/β-catenin signaling pathways, which subsequently led to progressive cardiomyocyte loss, fibrous tissue replacement, inflammatory infiltration, and cardiac impairment [12]. Both Akt and β-catenin pathways have been well established to be correlated with oxidative stress in cardiomyopathy, cardiac hypotrophy, and heart failure [13, 26, 35–37]. The Akt activation can further activate the Wnt/β-catenin pathway through the phosphorylation of GSK3-β [38]. Importantly, we found that the phosphorylation of Akt and protein levels of β-catenin in mouse heart tissue or in cardiomyocytes in vitro were promoted by ISO treatment, decreased by Mycn or USP2 overexpression, but restored after JUP knockdown. This evidence indicates that the Mycn/USP2/JUP axis suppresses the Akt/β-catenin cascade to exert anti-hypertrophic effects.

**Fig. 8:**
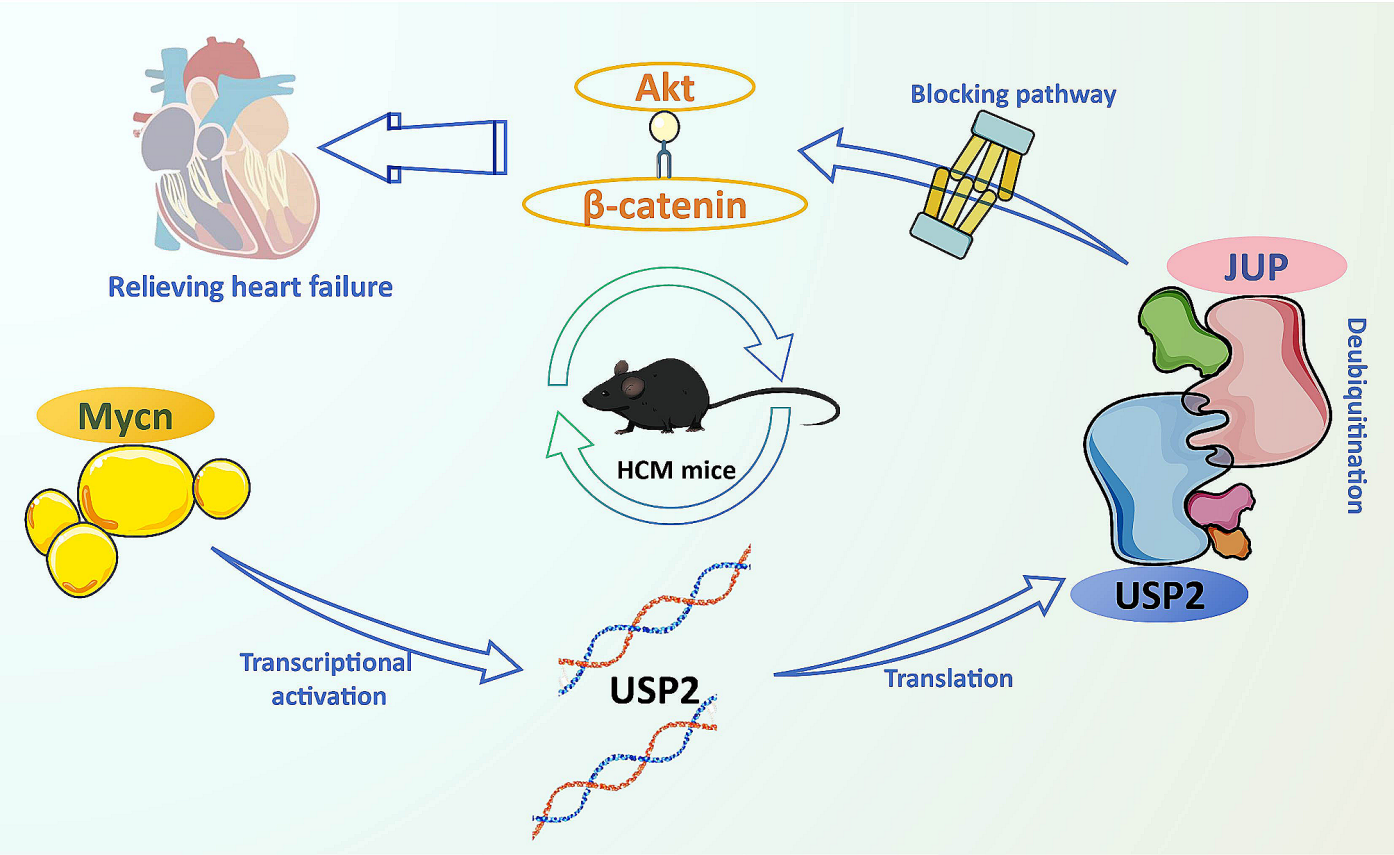
Graphical abstract. In the context of cardiac hypertrophy, Mycn ameliorates heart failure by activating transcription of USP2, which enhances the stability of JUP protein via deubiquitination modification and subsequently blocks the Akt/β-catenin pathway

In summary, this study provides novel evidence that Mycn ameliorates heart failure induced by cardiac hypertrophy. This effect is achieved through the activation of USP2, which enhances the stability of JUP protein via deubiquitination modification, subsequently blocking the Akt/β-catenin pathway (Fig. 8). This leads to reduced hypertrophic growth of cardiomyocytes, inflammation, and oxidative stress and cell death. Any members of the Mycn/USP2/JUP axis might serve as therapeutic tools for the management of HCM, though more in-depth investigations are required to validate our findings.

### Electronic supplementary material

Below is the link to the electronic supplementary material.

Supplementary Material 1

## Data Availability

The datasets used and/or analyzed during the current study are available from the corresponding author on reasonable request.
