# Supplementary material for: Mycn ameliorates cardiac hypertrophy-induced heart failure in mice by mediating the USP2/JUP/Akt/β-catenin cascade
Source: BMC Cardiovasc Disord. 2024 Jan 31;24:82. doi: 10.1186/s12872-024-03748-8 (PMC10829249; doi:10.1186/s12872-024-03748-8)

**Supplementary Figure S1** Original and full-length blot bands of c-CAS-3, Bcl-2, Bax, and IL-1β, corresponding to Fig 3C in the manuscript.


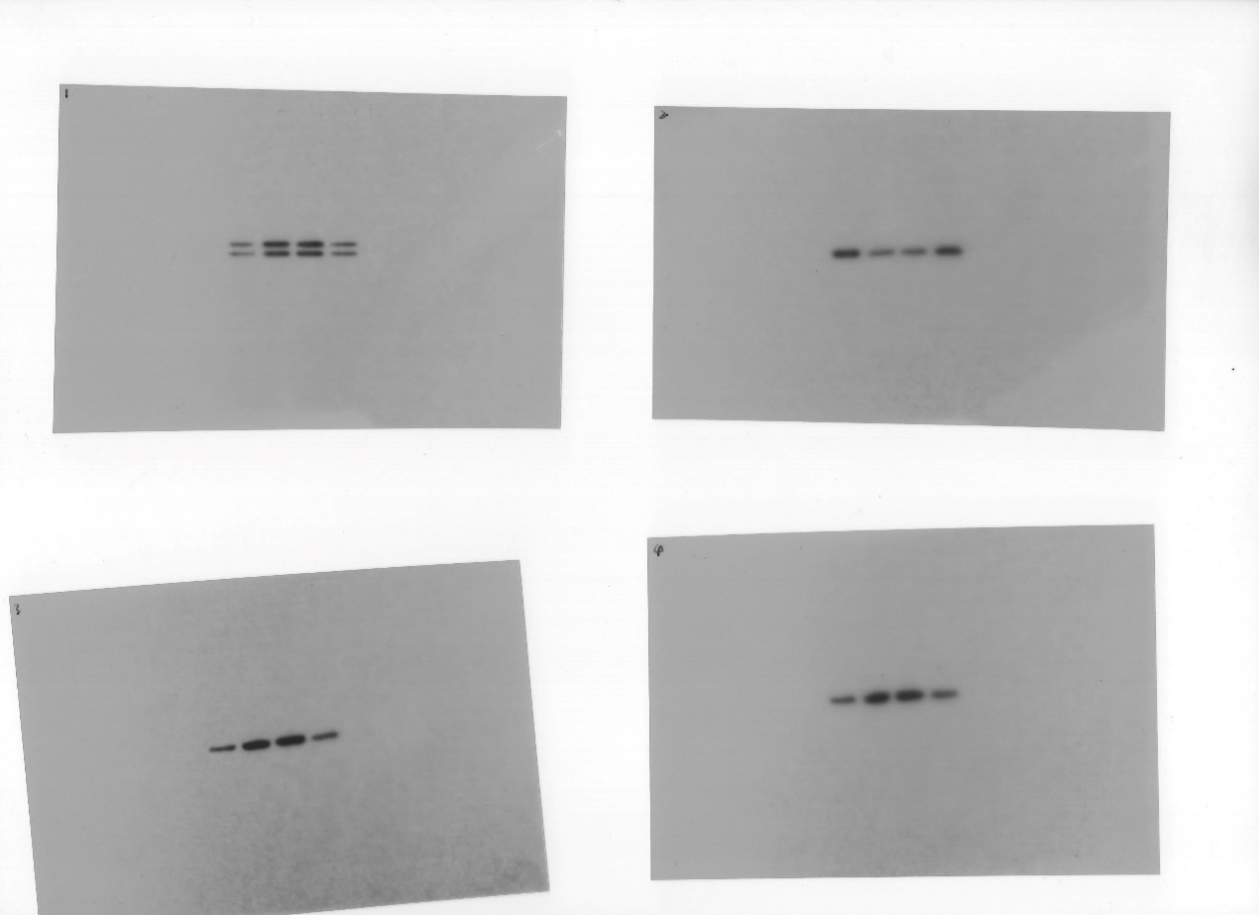


**Supplementary Figure S2** Original and full-length blot bands of TNF-α, MMP-9, and GAPDH corresponding to Fig 3C in the manuscript.


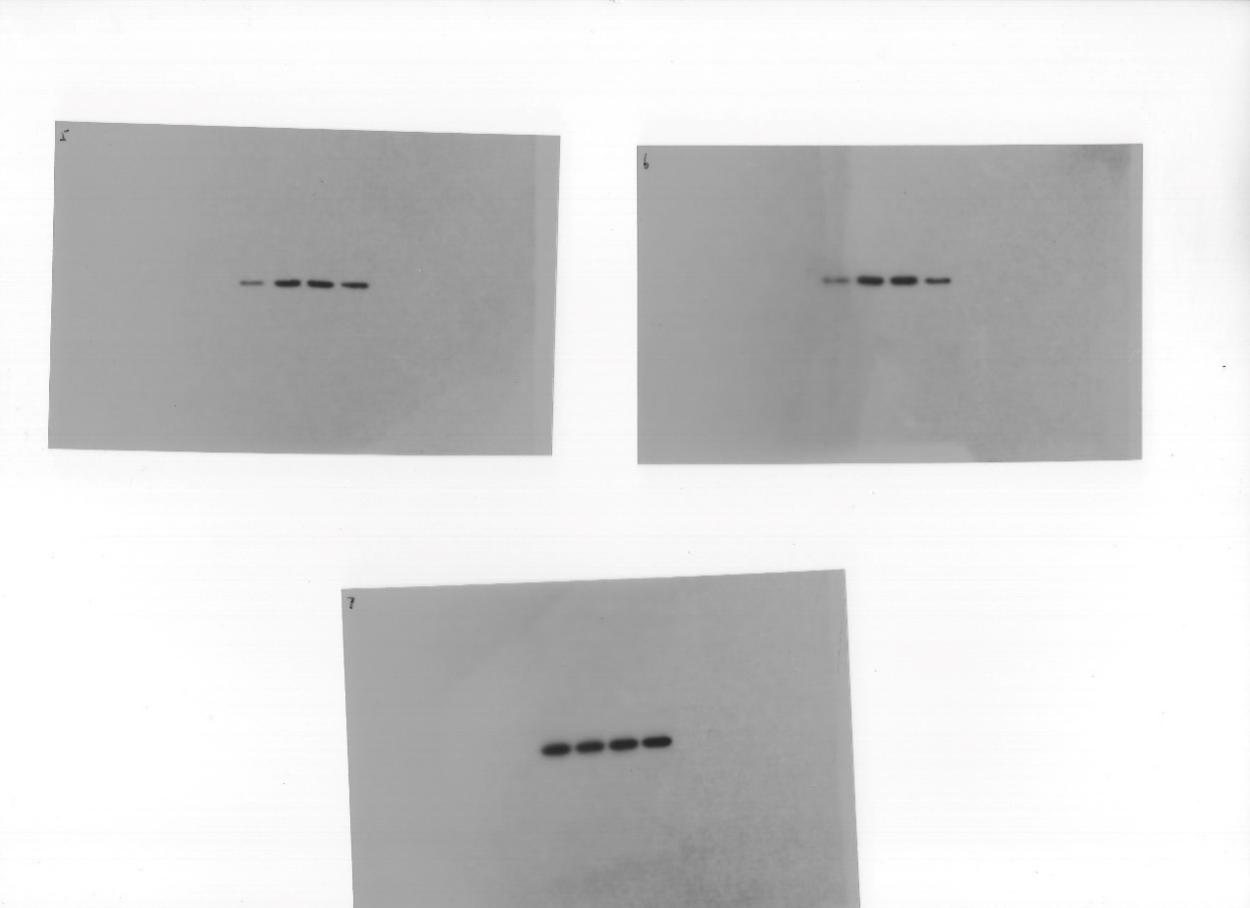


**Supplementary Figure S3** Original and full-length blot bands of USP2 and GAPDH, corresponding to Fig 4C in the manuscript.


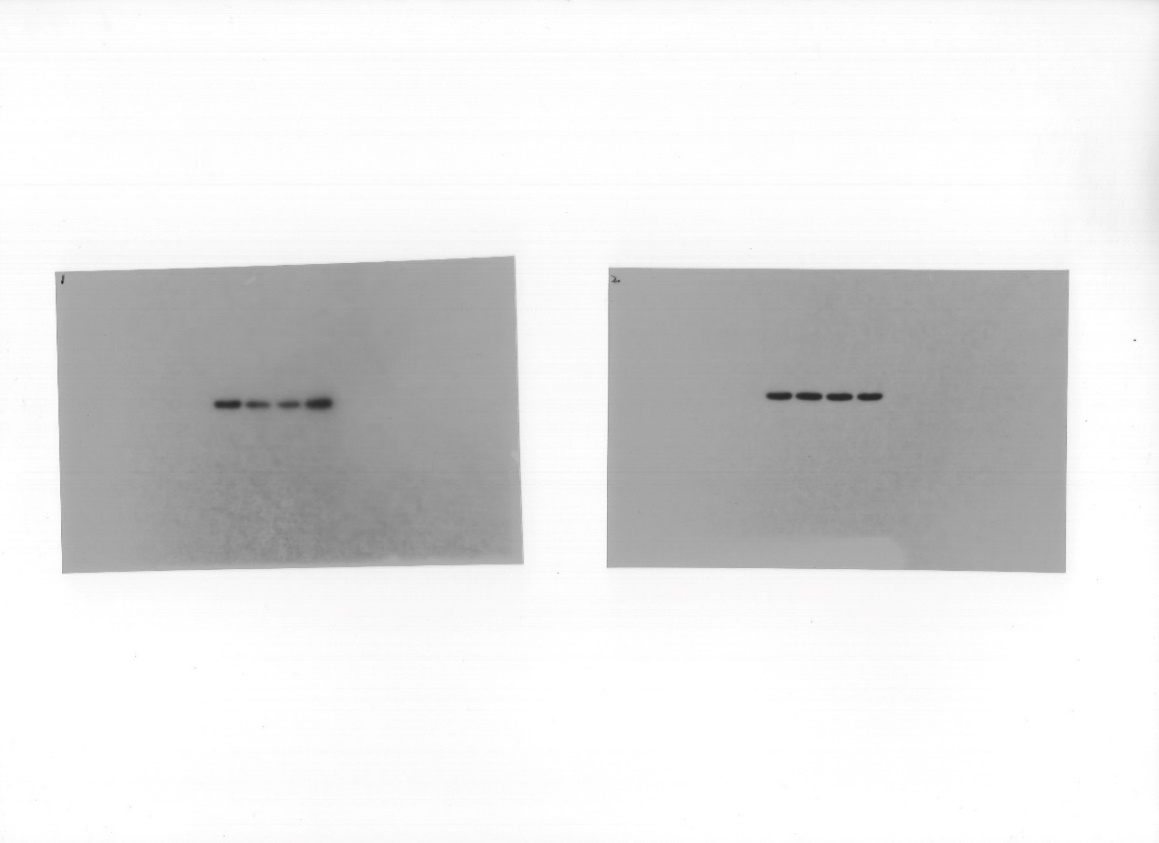


**Supplementary Figure S4** Original and full-length blot bands of JUP and GAPDH, corresponding to Fig 6B in the manuscript.
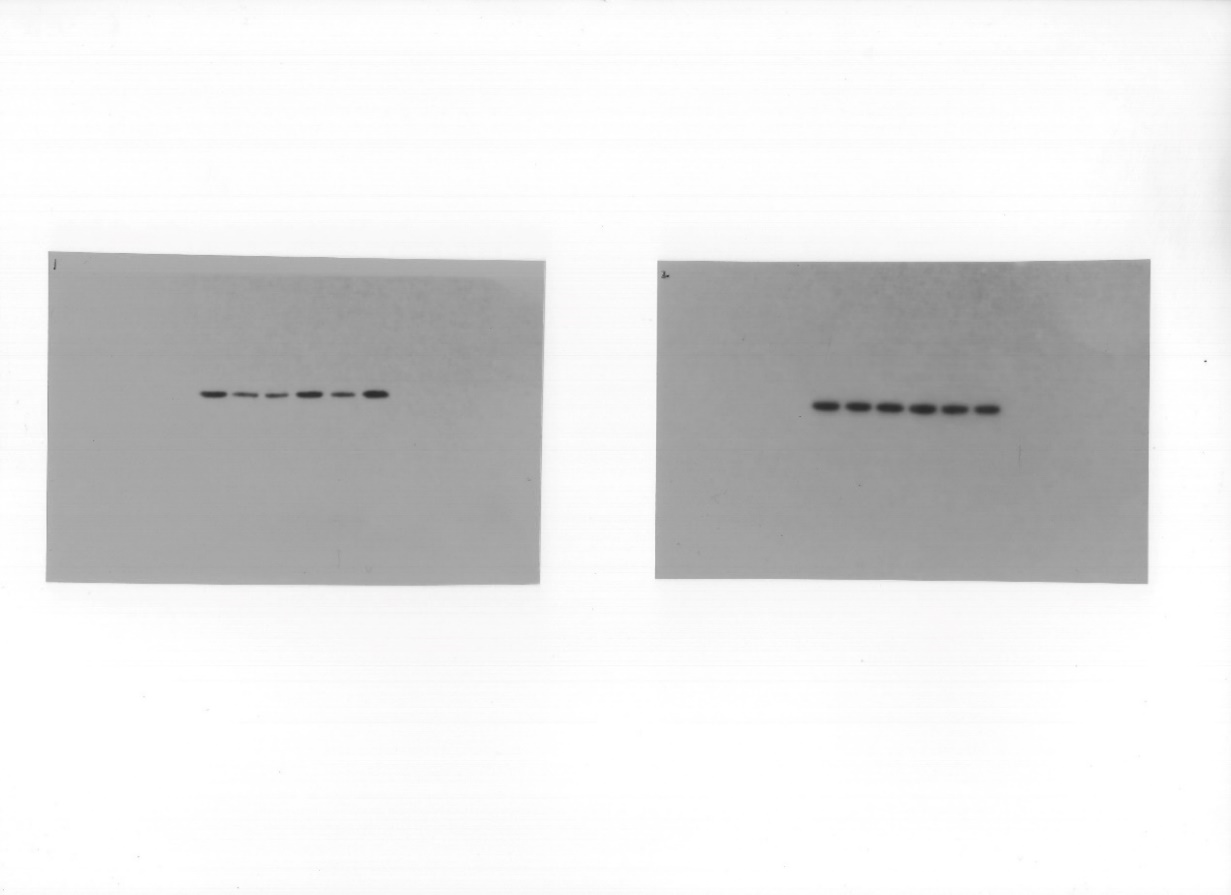


**Supplementary Figure S5** Original and full-length blot bands of USP2, JUP, and GAPDH, corresponding to Fig 6C in the manuscript.


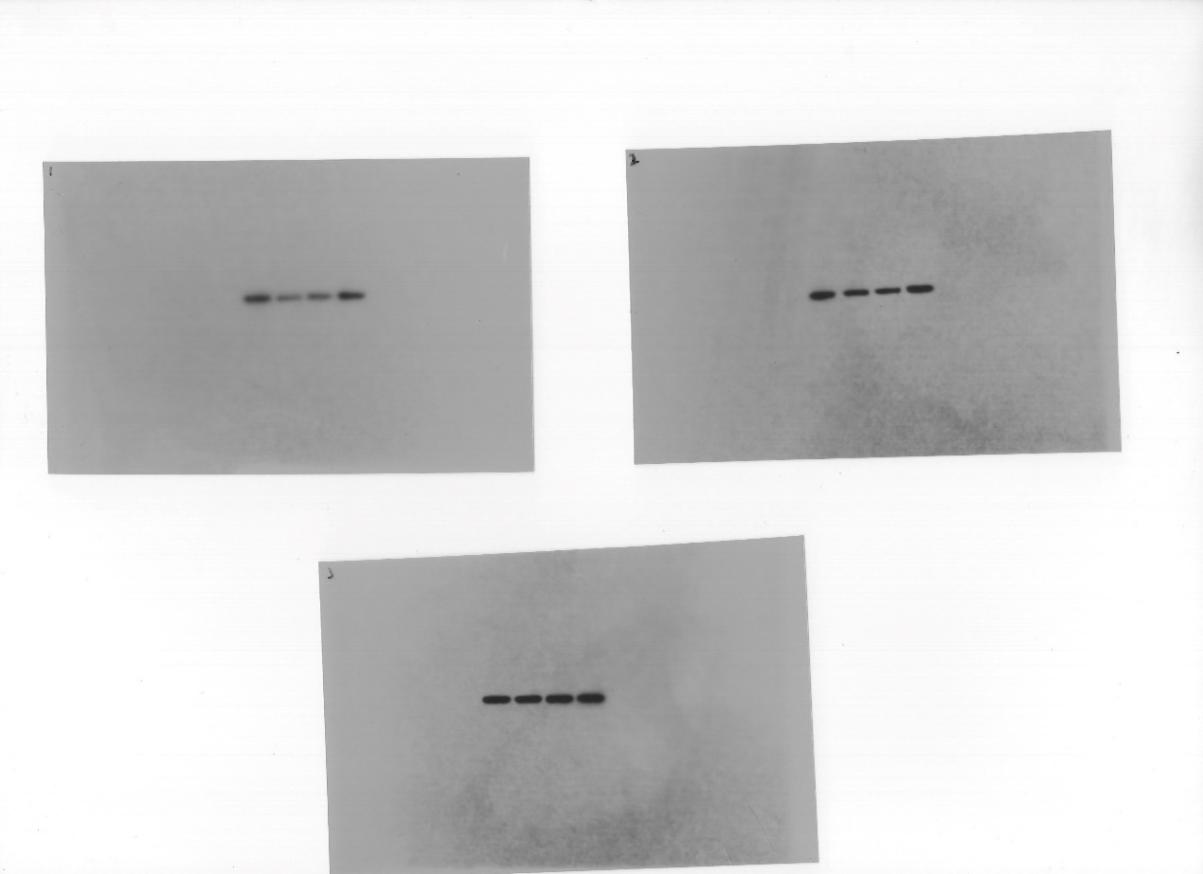


**Supplementary Figure S6** Original and full-length blot bands of Ub and USP2, corresponding to Fig 6D in the manuscript.


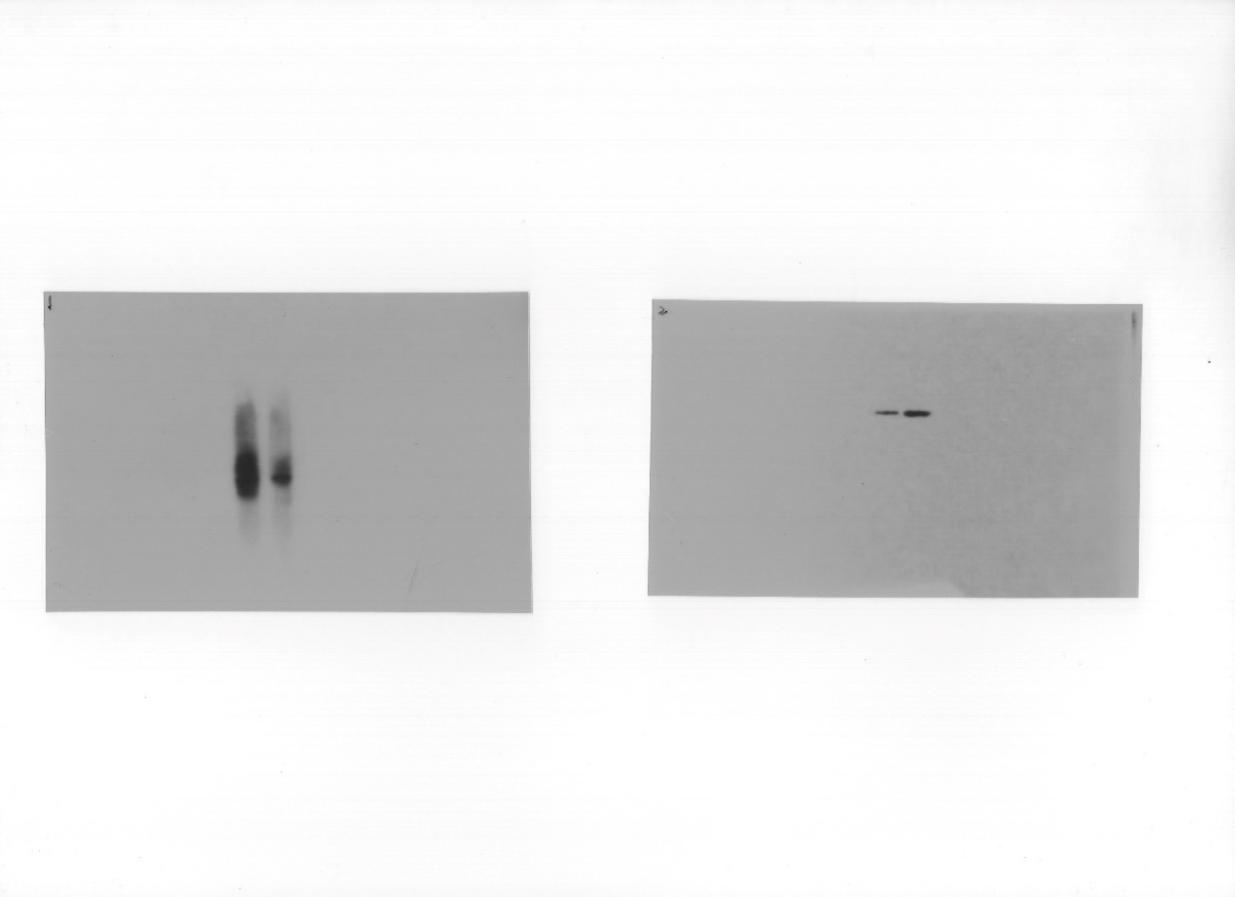


**Supplementary Figure S7** Original and full-length blot bands of USP2, JUP and GAPDH, corresponding to Fig 6D in the manuscript.


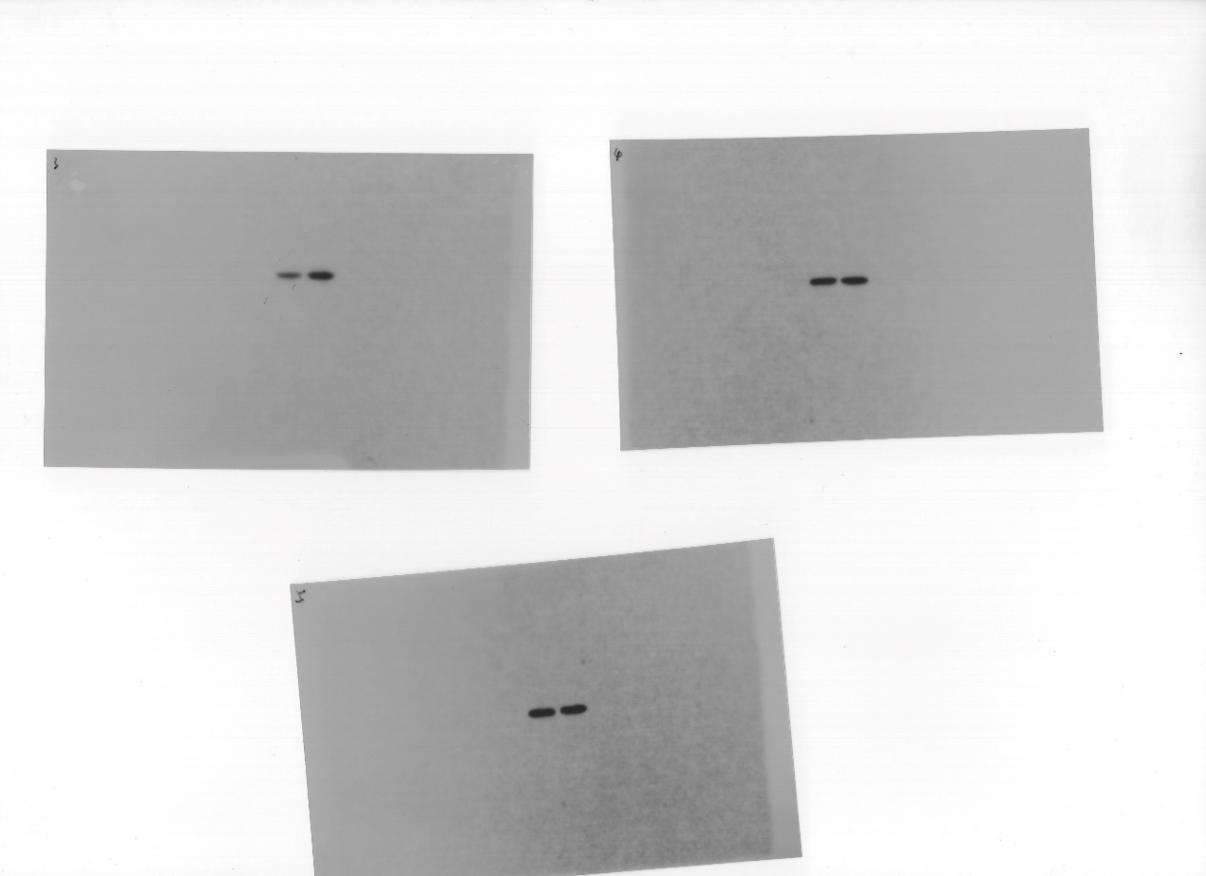


**Supplementary Figure S8** Original and full-length blot bands of JUP and GAPDH, corresponding to Fig 6E in the manuscript.


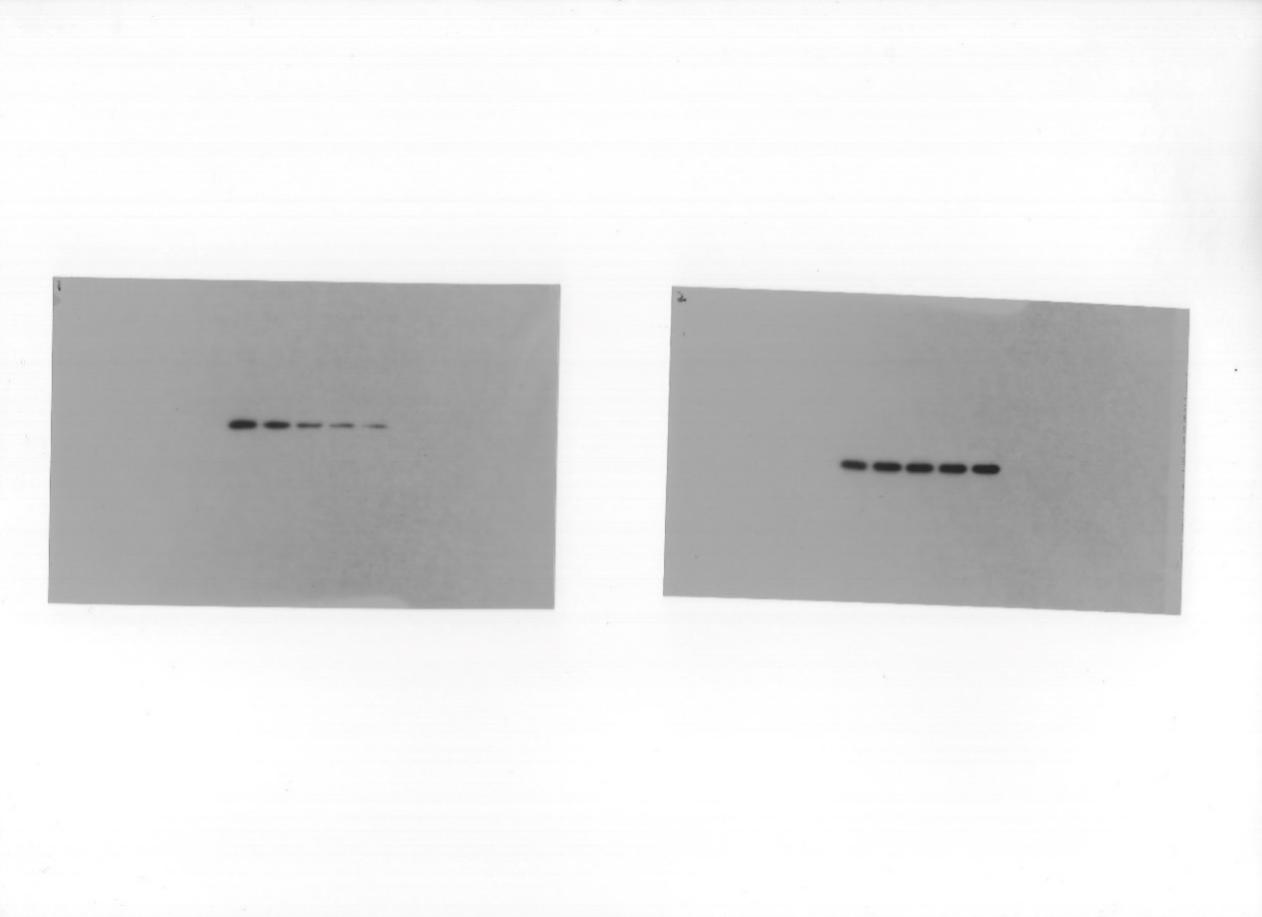


**Supplementary Figure S9** Original and full-length blot bands of JUP and GAPDH, corresponding to Fig 6E in the manuscript.


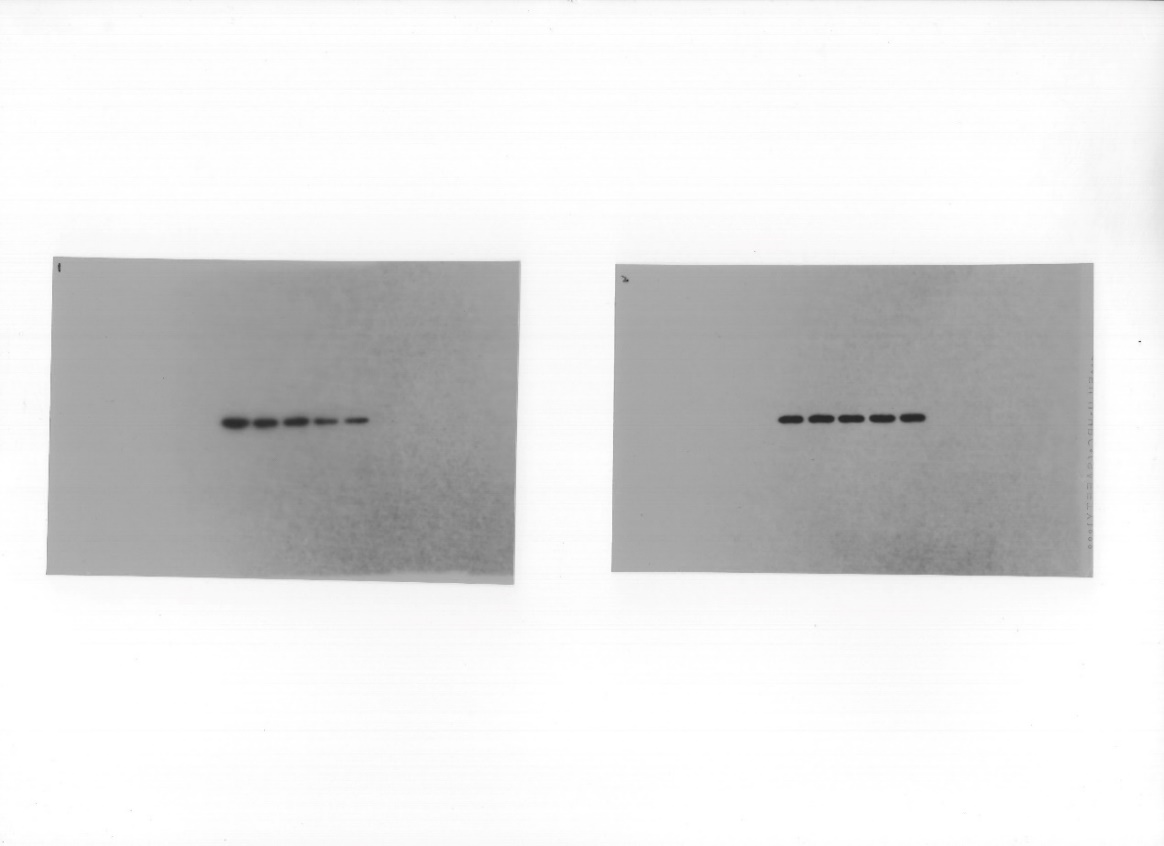


**Supplementary Figure S10** Original and full-length blot bands of p-Akt, β-catenin, and GAPDH, corresponding to Fig 6F in the manuscript.


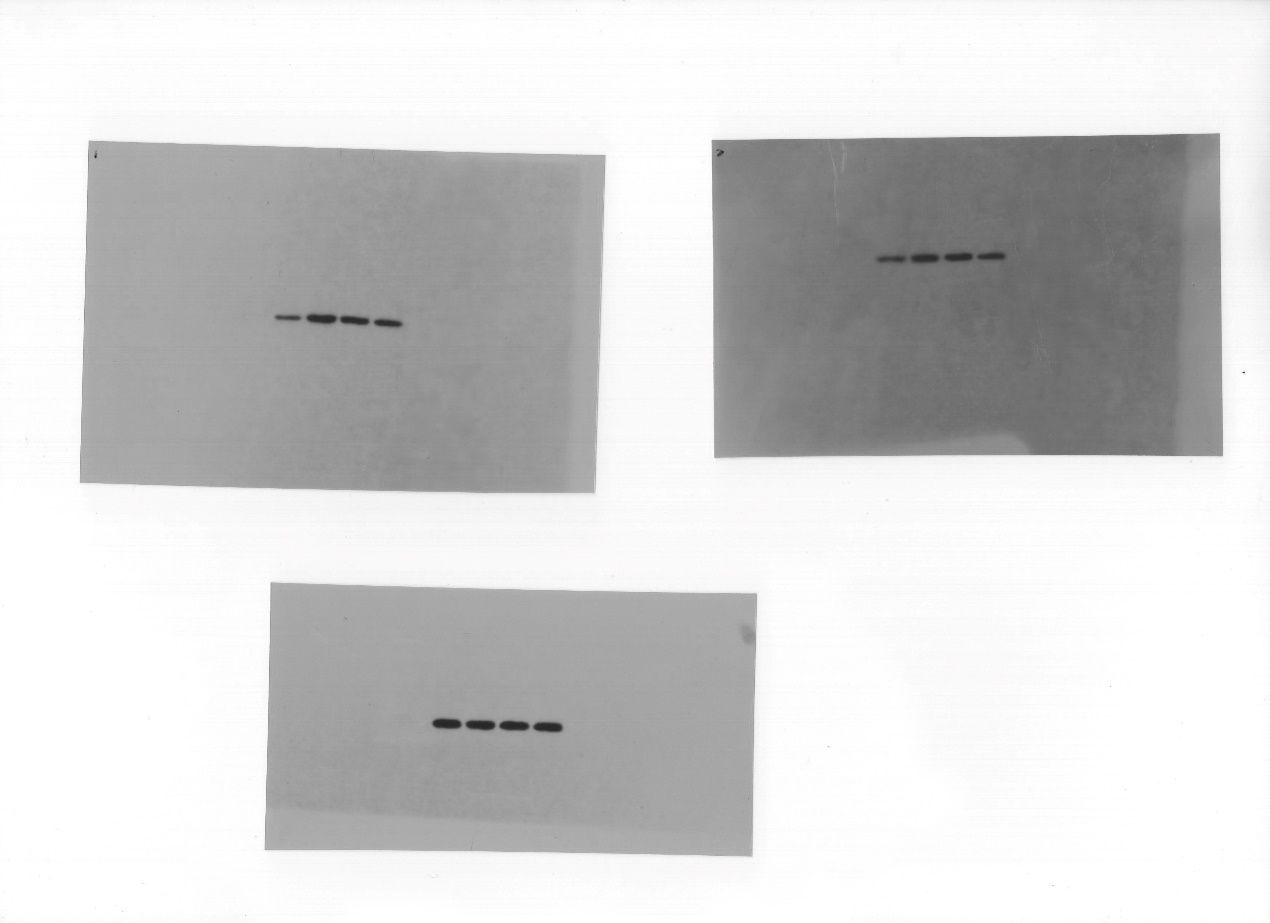


**Supplementary Figure S11** Original and full-length blot bands of p-Akt, β-catenin, and GAPDH, corresponding to Fig 6G in the manuscript.


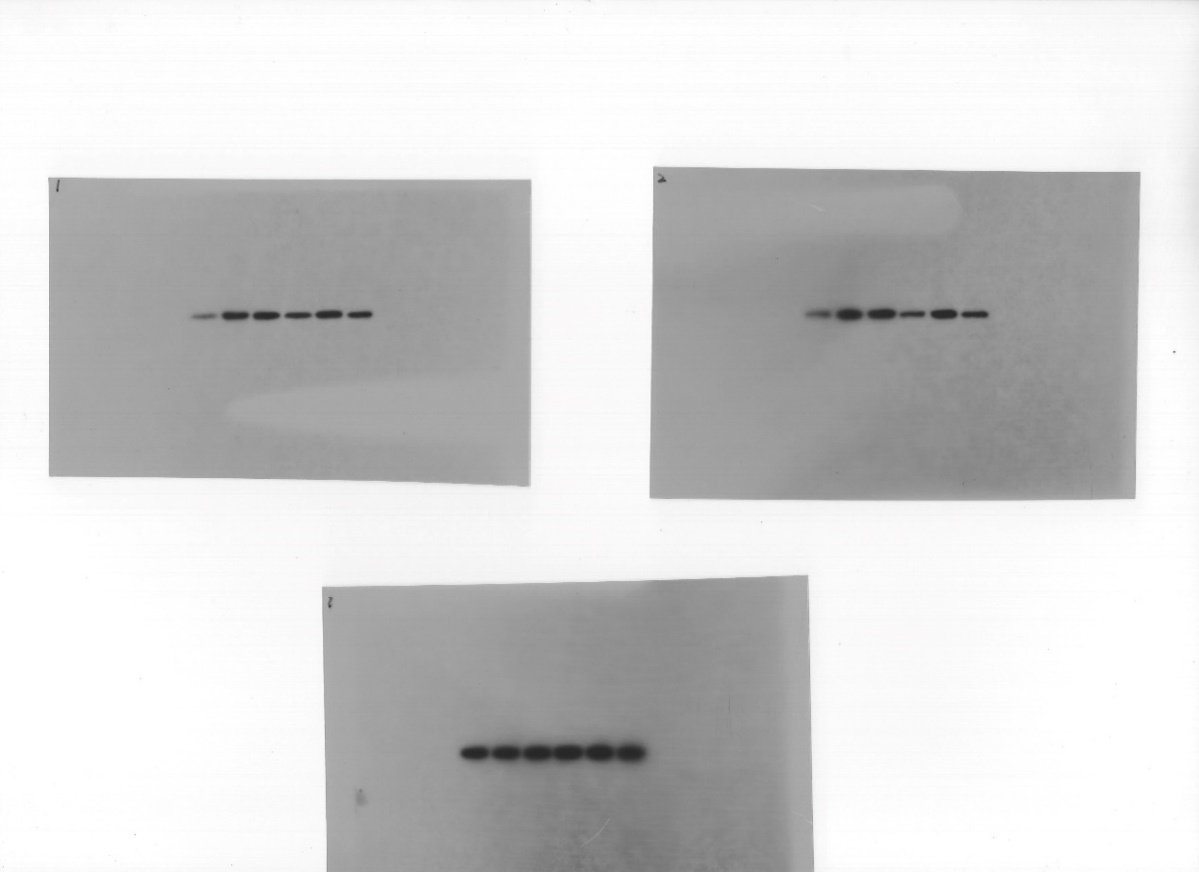


**Supplementary Figure S12** Original and full-length blot bands of p-Akt, β-catenin, and GAPDH, corresponding to Fig 7I in the manuscript.


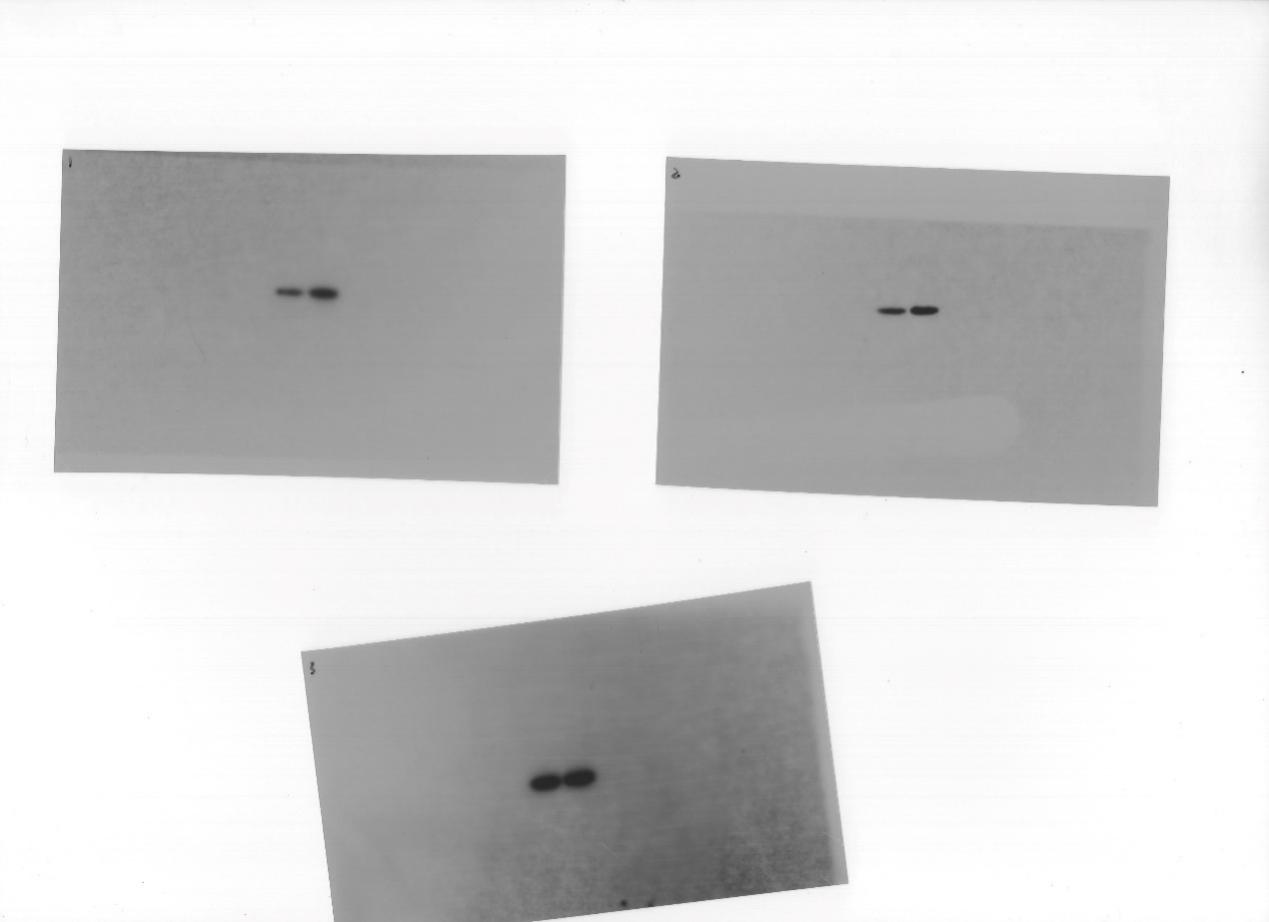

Supplement: Supplementary file 1 — Supplementary Material 1 [file 12872_2024_3748_MOESM1_ESM.docx]
